# A SARS-CoV-2 spike-derived adjuvant peptide boosts IL-17/IFN-γ immunity and improves anti-PD-L1 therapy against melanoma

**DOI:** 10.1186/s10020-025-01384-2

**Published:** 2025-12-27

**Authors:** Chia-Hung Chen, Tzu-Han Weng, Ta-Wei Kuo, Kai-Yao Huang, Yu-Chi Chen, Hsiao-Hsuan Huang, Hui-Ju Kao, Chen-Lin Yu, Chen-Chen Huang, Shun-Long Weng, Kuang-Wen Liao

**Affiliations:** 1 Department of Medical Research, Hsinchu MacKay Memorial Hospital, Hsinchu City, 30071 Taiwan, ROC; 2 Department of Medical Research, Hsinchu Municipal MacKay Children’s Hospital, Hsinchu City, 30068 Taiwan, ROC; 3 Department of Dermatology, MacKay Memorial Hospital, Taipei City, 10449 Taiwan, ROC; 4 Department of Biological Science and Technology, College of Engineering Bioscience, National Yang Ming Chiao Tung University, Hsinchu City, 30068 Taiwan, ROC; 5 Department of Medicine, MacKay Medical College, New Taipei City, 25245 Taiwan, ROC No.46, Sec. 3, Zhongzheng Rd. Sanzhi Dist.,; 6 Institute of Biomedical Sciences, MacKay Medical College, New Taipei City, 25245 Taiwan, ROC; 7 Industrial Development Graduate Program of College of Engineering Bioscience, National Yang Ming Chiao Tung University, Hsinchu City, 30068 Taiwan, ROC; 8 Department of Obstetrics and Gynecology, Hsinchu MacKay Memorial Hospital, Hsinchu City, 30071 Taiwan, ROC; 9 Nursing and Management, Mackay Junior College of Medicine, Taipei City, 11260 Taiwan, ROC; 10 Department of Obstetrics and Gynecology, Hsinchu Municipal MacKay Children’s Hospital, Hsinchu City, 30068 Taiwan, ROC; 11 Institute of Molecular Medicine and Bioengineering, College of Engineering Bioscience, National Yang Ming Chiao Tung University, Hsinchu City, 30068 Taiwan, ROC; 12 Center for Intelligent Drug Systems and Smart Bio-devices (IDS 2 B), National Yang Ming Chiao Tung University, Hsinchu City, 30068 Taiwan, ROC; 13 Department of Biotechnology and Bioindustry Sciences, National Cheng Kung University, Tainan City, 70101 Taiwan, ROC; 14 Graduate Institute of Medicine, College of Medicine, Kaohsiung Medical University, Kaohsiung City, 80708 Taiwan, ROC; 15 School of Dentistry, Kaohsiung Medical University School of Dentistry, Kaohsiung City, 80708 Taiwan, ROC; 16 Drug Development and Value Creation Research Center, Kaohsiung Medical University School of Dentistry, Kaohsiung City, 80708 Taiwan, ROC; 17 Department of Biological Science and Technology, National Yang Ming Chiao Tung University, Hsinchu City, Taiwan, ROC No.75 Po-Ai Street, 30068

**Keywords:** Severe acute respiratory syndrome coronavirus 2 (SARS-CoV-2), PD-L1, Bioinformatics analysis, Melanoma

## Abstract

**Background:**

PD-L1 immunotherapy plays a crucial role in cancer treatment, but PD-L1 peptide vaccines have low immunogenicity. A potent peptide derived from the spike protein of severe acute respiratory syndrome coronavirus 2 (SARS-CoV-2) has a significant adjuvant effect, which may increase the immunogenicity of the PD-L1 peptide. This study evaluates whether the PD-L1-SARS peptide enhances PD-L1 immunotherapy and analyzes its potential synergistic effects with anti-PD-L1 antibodies.

**Methods:**

In vivo experiments compared prevention, therapy, and combination therapy using PD-L1 versus PD-L1-SARS peptides in mice. Cytokine multiplex arrays, ELISpot, and IHC were used to evaluate adjuvant effects. Molecular docking (hypothesis-generating), RNA-seq, and LC–MS/MS were used to explore putative mechanisms.

**Results:**

The PD-L1-SARS peptide enhanced the Th1 immune response and increased CD8 and Th17 cell infiltration, effectively inhibiting tumor growth and liver metastasis. Additionally, it promoted M1 macrophage polarization and improved anti-PD-L1 antibody efficacy. Proteomics and bioinformatic analyses were consistent with IFN-γ–linked pathways, and an exploratory docking screen nominated candidate receptors/pathways potentially connecting the adjuvant motif to innate sensing.

**Conclusions:**

Embedding a SARS-derived adjuvant-like motif within a PD-L1 peptide vaccine and delivering it in situ may re-condition the tumor microenvironment toward an immune-activating, Th1/Th17-biased state and complement PD-L1 blockade.

**Graphical abstract:**

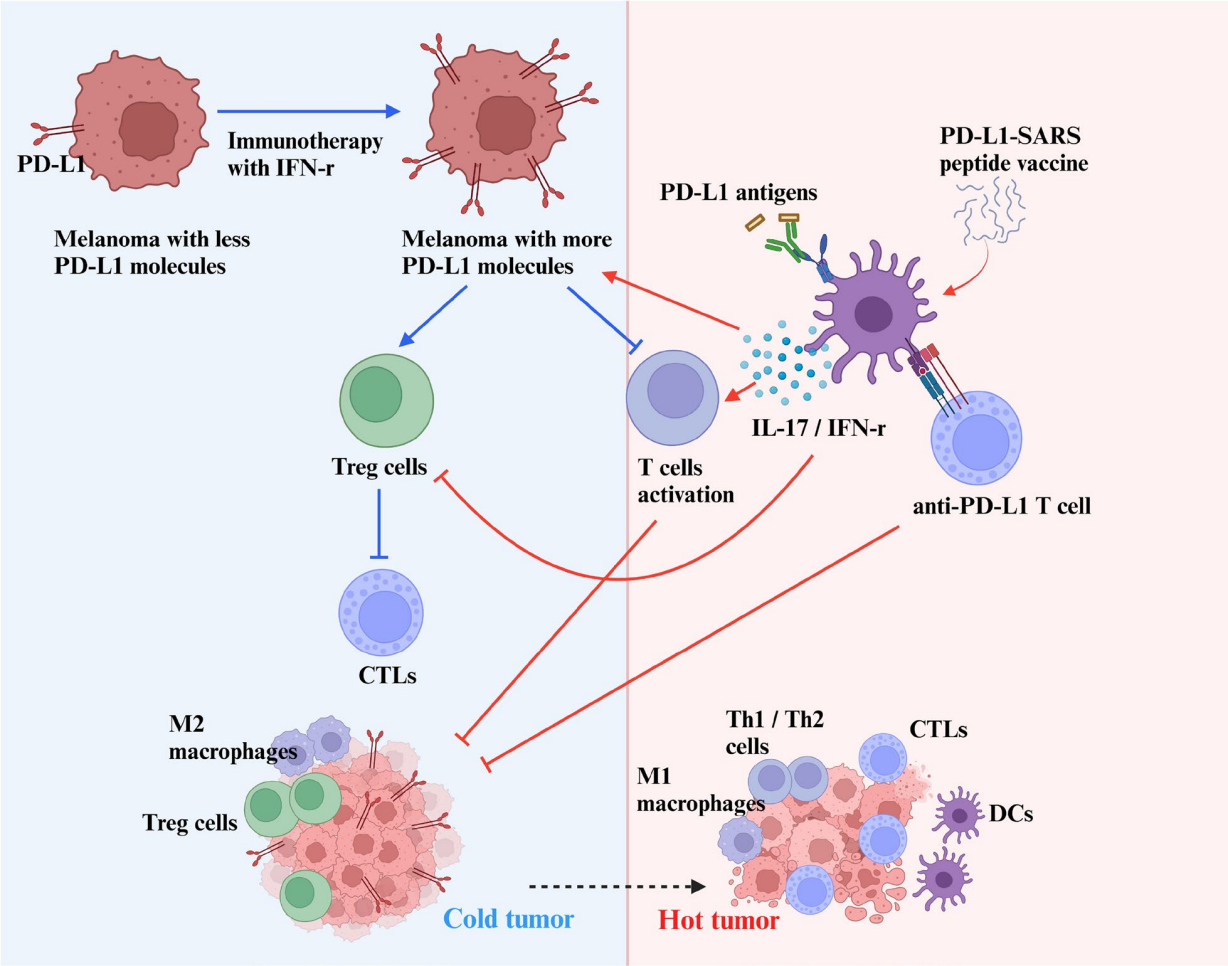

**Supplementary Information:**

The online version contains supplementary material available at 10.1186/s10020-025-01384-2.

## Background

Tumor vaccines show promise in cancer treatment; however, efficiently inducing proper antitumor immune responses remains a significant challenge [1, 2]. First, the use of whole proteins as tumor antigens usually leads to the induction of antibodies with different epitopes for an antigen. It is worse if the antibodies can induce tumor growth. On the contrary, if the antibodies have a character of blocking activity against the proteins of normal cells, it may cause cell dysfunction or autoimmune diseases after a certain period of treatment [2]. This underscores the urgency and importance of peptides derived from tumor antigens and their potential implications for cancer treatment.

Peptide-based tumor vaccines are typically composed of amino acid sequences derived from tumor-specific antigens (TSAs) or tumor-associated antigens (TAAs). To be efficacious, such peptide sequences must contain CD8 epitopes to activate cytotoxic T lymphocyte (CTL)-mediated antitumor immunity, along with CD4 epitopes for T-helper cell activation, which sustains CTL effector function [3, 4]. However, the immunogenicity of these TAA-derived peptide antigens is usually too weak to elicit sufficient immune responses against tumor cells [5, 6]. Therefore, adjuvants are essential for enhancing peptide-induced antitumor immunity. Cytokines, such as granulocyte-macrophage colony-stimulating factor (GM-CSF) [7, 8] or IL-12 [9, 10], have been utilized to meet this expectation and have shown promising results.

Viral proteins are known to trigger the expression of cytokines and can act as adjuvants [11]. SARS-CoV-2 infection causes significant inflammation through innate immune cells such as macrophages, monocytes, and neutrophils. These cells are activated in the presence of excessive amounts of inflammatory cytokines and chemokines [12, 13]. This mechanism is induced by the binding of the SARS-CoV-2 spike (S) protein to TLR2, which stimulates the NF-κB pathway and causes the release of inflammatory cytokines and chemokines [14]. Another study revealed that the S protein also induces inflammatory cytokine secretion via the JNK pathway in murine macrophages [15]. Therefore, the S protein may have potential as an adjuvant for vaccinating low-immunogenicity peptides derived from tumor antigens.

Melanoma is one of the most malignant skin cancers and is characterized by early metastasis, rapid development, a poor prognosis, and a high mortality rate [16]. PD-L1 molecules, which are usually expressed in many tumor cells, trigger the apoptosis of activated T cells or inhibit their activities [17, 18]. In melanoma, PD-L1 expression is most often observed on malignant melanocytes [19]. Blockade of the PD-L1 molecule enhances the T cell antitumor response and improves clinical outcomes [20]. The literature indicates that the existence and activity of anti-PD-L1 T cells are capable of helpful tumor therapeutic effects in patients [21–24]. Therefore, strategies for inducing an anti-PD-L1 Th1/Th2 cell-mediated immune response should be considered.

Prior PD-L1–targeted peptide vaccines typically rely on external carriers/adjuvants and still struggle to convert “cold” tumors into productive cellular immunity. SARS-derived short motifs have been explored as innate immune stimuli, but embedding such a motif within a PD-L1-directed peptide and delivering it in situ to re-condition the tumor microenvironment (TME) remains underexplored. Here we introduce a PD-L1 peptide vaccine that incorporates a SARS-derived adjuvant-like motif and is administered in situ, aiming to couple antigenic specificity with local innate priming. We position this concept relative to traditional carriers/adjuvants, checkpoint antibodies, and transmembrane-domain (TMD)-targeting chemotypes, and we outline an in silico, hypothesis-generating receptor screen to guide future mechanistic validation. Therefore, in this study, on the basis of the characteristics of malignant melanoma, vaccines were designed to target surface makers that are overexpressed in order to activate antitumor and humoral immunity [25]. We constructed a fusion peptide by fusing a partial sequence of PD-L1, with a highly immunogenic fragment from the SARS S protein which has been verified to effectively elicit IFN-γ secretion in humans [26] and to increase Th1 and Th2 cytokine secretion in mice [27]. Herein, the experimental results showed that this fusion peptide significantly retarded tumor growth and liver metastasis in mice. Besides, it synergistically increased the therapeutic efficacy of anti-PD-L1 antibodies. Bioinformatic analysis speculated that, mechanistically, the SARS peptide targets multiple molecules induce IL-17 and IFN-γ expression to activate the anti-PD-L1 T-cell response against melanoma cells. This peptide vaccine platform is a promising strategy to activate specific antitumor immunity by adjuvant effect on low-immunogenicity peptides derived from tumor antigens. And the fusion peptide was delivered in situ to address two unmet needs of PD-L1 peptide vaccination: limited cellular immunogenicity and suppressive TMEs. This approach is conceptually distinct from carrier-only vaccines and checkpoint antibodies, and complementary to TMD-targeting chemotypes.

## Methods

### Selection of PD-L1 and SARS peptides

The amino acid sequence and domain information of mouse PD-L1 were obtained from the UniProt database (www.uniprot.org). The extracellular domain (a.a. 19–239) was loaded in the IEDB database (www.iedb.org) and assessed to determine the most suitable epitopes of C57BL/6J mouse MHC-I (H-2Kb) and -II (I-Ab). A fragment of the SARS spike glycoprotein had been verified to efficiently induce IFN-g secretion in a mouse model [11]. Similarly, the epitope was obtained as described above. Table S1 shows that the PD-L1 sequence was ELIIPELPATHPPQNRTSNFRGRASL, and the PD-L1-SARS sequence was ELIIPELPATHPPQNRTAAPHGVVFLHVTYVSNFRGRASL. The PD-L1 and PD-L1-SARS peptides were each coupled with KLH carrier proteins for mouse immunization.

### Anti-PD-L1 peptide antibodies in serum

Female C57BL/6J mice (4–5 weeks old) were divided into four groups, and each group was immunized via subcutaneous injection with 100 µL of PBS, 100 µg of KLH, 100 µg of KLH-PD-L1, or 100 µg of KLH-PD-L1-SARS. Peptide vaccines were formulated in PBS; when DMSO was required for solubilization, the final DMSO concentration was ≤ 2% (v/v), with a 100 µL injection volume. The mice were boosted every two weeks after immunization for a total of 12 weeks. In addition, blood was collected by cheek bleeding, and the titer of PD-L1-specific antibodies in the serum samples was determined. The sera (1:8000 dilution) was evaluated. Similarly, the antibody isotypes in the sera of the mice were measured.

Serum samples were collected in the 12th week of the vaccination process, and ten microliters of serum was incubated with 2 × 10^5^ B16F10 cells at 4 °C for 1 h. The binding activity of the anti-PD-L1 antibody was determined by BD Accuri™ C6 flow cytometry (BD Biosciences, San Jose, CA, USA).

### Cytokine array

The mice were vaccinated as described above. Serum samples were collected and analyzed at a tenth-week time point during the vaccination process. Besides, these mice splenocytes were isolated and seeded at 1 × 10^6^ cells/well in a 24-well culture plate and then treated with 0.1 µg/mL different peptides for 72 h. The sera or supernatants in each group were collected for analysis of cytokine levels via a cytokine multiplex assay using the Bio-Plex Pro Mouse Cytokine Standard Group I 23-Plex following the manufacturer’s protocol (Bio-Rad, Hercules, CA, USA).

### CTL assay

B16F10 cells were seeded in a 96-well plate (1 × 10^3^ cells/well) and incubated overnight. Mouse splenocytes isolated from different treatment groups were added to the wells as effector cells at E: T ratios of 5:1, 10:1, and 20:1. After incubation for 72 h, the supernatants were collected, and the degree of cell cytotoxicity was assessed by using the CyQUANT™ LDH Cytotoxicity Assay Kit according to the manufacturer’s protocol (Sigma-Aldrich).

### ELISpot

Mice were immunized and boosted with KLH-PD-L1-SARS or others as described above. To evaluate the immune response, splenocytes were harvested from treated mice after immunization. Prior to analysis, the isolated splenocytes (1 × 10^6^ cells/per well) were pulsed with the SARS peptide antigen (1 µg/mL) to stimulate antigen-specific responses. Following stimulation, the number of cells expressing IL-4 or IFN-γ was quantified using a Mouse IFN-gamma/IL-4 Dual-Color ELISpot Kit (R&D Systems, MN, USA), according to the manufacturer’s protocol. Ressulting spots were visualized and counted under an inverted microscope (Olympus, Shinjuku, Tokyo, Japan). ELISpot spots were counted blinded, background-subtracted, and expressed as spot-forming cells (SFCs)/10⁶ cells; analyses included the IFN-γ/IL-4 ratio to summarize Th1/Th2 balance.

### Tumor growth prevention

As described above, when the mice were immunized with KLH-PD-L1-SARS for 12 weeks, female C57BL/6J mice (4–5 weeks old; *n* = 7–9 per group) were subcutaneously implanted with 1 × 10^4^ B16F10 cells in 100 µL of PBS. To facilitate reproducible caliper measurements, the fur over the right dorsal flank was clipped immediately before tumor inoculation (the sparse hair visible in images reflects this planned clipping, not spontaneous alopecia). Tumor size was measured with a caliper, and the tumor volume was calculated as L × H × W × 0.5236. The mice were sacrificed when the tumor size exceeded 1,000 mm^3^. Additionally, body weight was recorded at each measurement. Local reactogenicity (erythema/induration) was also monitored.

### H&E and immunohistochemistry staining

H&E and immunohistochemistry (IHC) staining analyzed the tumors and organs according to the previous study [28]. The tissue slides were photographed to observe the pathology and metastatic colonies under a microscope (Olympus, PA).

Antigen Retrieval Buffer (Abcam, Cambridge, UK) and a Leica Novolink max polymer detection system (RE7140-K, Wetzlar, Germany) for IHC staining were used following the manufacturer’s protocols. Finally, the sections were probed with specific commercial antibodies (Table S2), and the samples were observed under a light microscope and photographed.

### In situ tumor therapy

Female C57BL/6J mice (4–5 weeks old; *n* = 8–10 per group) were subcutaneously inoculated with 1 × 10⁴ B16F10 cells in the right dorsal flank. To facilitate reproducible caliper measurements, the fur over the right flank was clipped immediately before tumor inoculation. Tumor growth was monitored, and when an individual tumor reached ~ 30 mm³ (pre-specified 20–40 mm³ window), animals were block-randomized stratified by baseline tumor volume; the day of randomization was defined as Day 0. Beginning on Day 0, mice received weekly intratumoral (in situ) injections of the peptide vaccine (100 µg per dose) according to group assignment. Tumor volume was then measured every two days. Where applicable, tumor measurements were performed by an investigator blinded to allocation, and mice were euthanized when tumor volume exceeded 1,000 mm³ or if other humane endpoints were met.

### Combination therapy against tumors

All procedures for tumor inoculation, fur clipping, randomization, Day-0 definition, tumor measurement schedule, volume calculation, blinding, and humane endpoints were as described above. Briefly, when individual tumors reached ~ 30 mm³ (20–40 mm³ window), mice (*n* = 10 per group) were block-randomized and treatment was initiated (Day 0). Beginning on Day 0, animals received weekly intratumoral peptide vaccine (100 µg) and anti-mouse PD-L1 antibody (clone 10 F.9G2, Bio X Cell) intraperitoneally at 10 mg/kg every two days according to group assignment. Tumor volumes were measured every two days.

### Docking of the SARS-derived peptide (hypothesis-generating)

We implemented a peptidomimetic-guided, reverse-screening workflow to contextualize potential receptor engagement for the SARS-derived peptide. The 14-aa sequence was partitioned into overlapping 6-aa subfragments (sliding window). For each subfragment, side-chain topology/pharmacophore features were mapped to small-molecule–like surrogates (SMILES) and queried against BindingDB (https://www.bindingdb.org) via structure/similarity search to retrieve published experimental binding affinities of literature small-molecule ligands for the corresponding protein targets.

Importantly, these BindingDB affinities pertain to small molecules, not our peptide. In our dataset, the provider reported affinities as inverse values (e.g., 1/Kd in nM⁻¹); when values were available as Kd/Ki/IC₅₀ in nM, we inverted them so that larger numbers correspond to stronger affinity. To enable unitless visualization for each fragment–target pair, these affinity-derived numbers were converted to a Predicted binding index (unitless). This index is used descriptively to indicate predicted interaction propensity for each subfragment with each candidate protein; no statistical ranking/thresholding is applied, and the index does not represent experimental affinity of the peptide nor imply causality.

Independent protein–peptide docking was performed with HDOCK (http://hdock.phys.hust.edu.cn) [29] using default parameters to visualize poses and interface residues (peptide–protein residue pairs within 3.5 Å). Docking outputs were used qualitatively to support candidate contextualization and were not converted to experimental affinities. Binding confidence was categorized as follows: confidence scores above 0.7 indicated a high likelihood of binding; scores between 0.5 and 0.7 suggested possible binding; and scores below 0.5 indicated that binding was unlikely.

### RNA sequence analysis of mouse splenocytes

Naïve mouse splenocytes were treated with PD-L1 or PD-L1-SARS peptides for five days, which was according to the RT-PCR results (Fig. S1A), and splenocyte RNA was extracted using a PureLinkTM RNA Mini kit (Thermo Fisher Scientific Inc., Waltham, MA). NGS was subsequently performed with a NovaSeq 6000 system (Illumina, CA). The raw counts were estimated and normalized as transcripts per million (TPM) values. Gene expression was further analyzed via *t*-test and ANOVA. The genes with significant differences (*P* < 0.01) through *t*-test, and then the 851 genes occupying the top 70% of the expression amount were selected for subsequent CIBERSORTx, CD marker and immune cytokine expression analyses (Fig. S1B). All supporting NGS data were available to researchers in the Gene Expression Omnibus (GEO) database, and the accession number was PRJNA1007364.

### LC‒MS/MS analysis of tumor tissues under in situ treatments

Female C57BL/6J mice (*n* = 4) were inoculated subcutaneously with 1 × 10^4^ B16F10 cells. When the average tumor volume in each group reached 50 mm^3^, the mice were injected with 100 µg of peptide vaccine in situ. After seven days, the tumor tissues were collected for LC‒MS/MS analysis. The raw files were searched against a UniProt mouse database via the Sequest and Mascot search engines in Proteome Discoverer (version 2.5).

### Statistical analysis

Numeric data were expressed as the mean ± SD. Data were analyzed using the SAS statistical package (SAS Institute, Inc., Cary, NC, USA). A Student’s *t*-test was used when comparing two independent samples, and ANOVA was used for comparing multiple samples. *P* < 0.05 was considered to indicate statistical significance.

## Results

### Immunogenicity of the PD-L1-SARS peptide vaccine

Melanoma cells overexpress PD-L1 on their surface; therefore, PD-L1 is considered a tumor-associated antigen. Furthermore, T-cell epitopes in PD-L1 were determined through computational analysis using the IEDB database, as antigens. Similarly, the peptide sequences were derived from the SARS-CoV-2 spike protein as an adjuvant, and these peptide sequences were shown in Table S1.

**Fig. 1:**
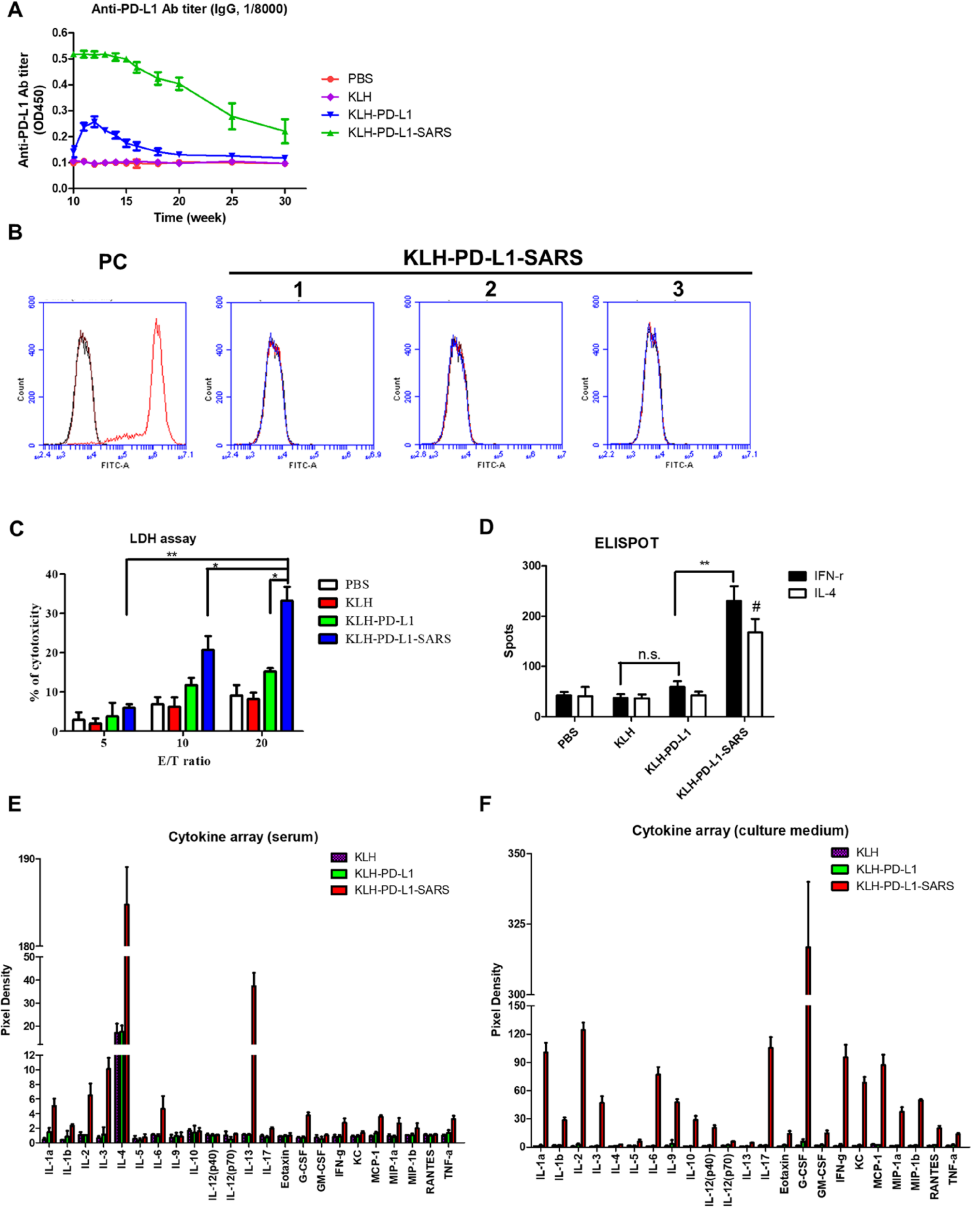
**The immunogenicity of the PD-L1-SARS peptide vaccine.** **A** Time evaluation of the anti-PD-L1 antibody titer of the KLH-PD-L1-SARS vaccine. **B** Three mice serum antibodies immunized with KLH-PD-L1-SARS could not recognize PD-L1 molecules on the cell surface. **C** The cytotoxicity of splenocytes immunized with KLH-PD-L1-SARS against B16F10 cells had a dose-dependent manner. A significant difference was indicated by * (*P* < 0.05) and ** (*P* < 0.01). Data were expressed as the mean ± SD (*n* = 6). **D** KLH-PD-L1-SARS efficiently increased the cell numbers of IFN-γ and IL-4-expressing T cells; a Th1/Th2 balance index (IFN-γ/IL-4) is reported in the text. A significant difference was indicated by ** (*P* < 0.01), and a comparison with the KLH-PD-L1 group is indicated by # (*P* < 0.05). Data were expressed as the mean ± SD (*n* = 6). **E** and **F** Cytokine profile expression of immune cells derived from vaccinated mice. In both serum and culture medium, several cytokines were highly expressed in the KLH-PD-L1-SARS group but not in the other groups

After immunization with the different peptide vaccines, antibody titers and recognized activity were determined against peptide antigens or the cell surface antigen. The data indicated that KLH-PD-L1-SARS could dramatically induce the production of anti-PD-L1 antibodies against peptides, while KLH-PD-L1 had a minor effect (Fig. 1A). However, the result showed that the sera antibodies of all vaccinated mice did not recognize the PD-L1 antigen on the cell surface (Fig. 1B).

The cytotoxicity assay results indicated that splenocytes from mice immunized with KLH-PD-L1-SARS exhibited greater dose-dependent cytotoxicity than did those from the control groups (Fig. 1C). Although immunization with KLH-PD-L1 peptides resulted in a slight increase in cytotoxicity, this increase was not statistically significant. ELISpot showed that KLH-PD-L1-SARS increased PD-L1–specific IFN-γ and IL-4 SFCs (per 10⁶ cells, background-subtracted) versus controls (Fig. 1D). To summarize polarization, we computed a Th1/Th2 balance index (IFN-γ/IL-4; mean of individual ratios), which was 1.39 ± 0.08 (*n* = 6), indicating a Th1-leaning cellular response.

Changes in cytokines and chemokines in the blood and lymphoid organs (spleen) were monitored. Compared with other treatments, immunization with KLH-PD-L1-SARS efficiently led to higher expressions of proinflammatory cytokines in the blood (Fig. 1E). Unlike the systemic effect, immunization with KLH-PD-L1-SARS increased the IL-17 and IFN-γ levels in cultured immunized splenocytes. Additionally, it promoted the production of G-CSF. Furthermore, these immune cells secreted inflammatory cytokines (Fig. 1F). These results indicated that this SARS-CoV-2 peptide exhibited a potent adjuvant effect, which could promote immune cells to release proinflammatory cytokines, IL-17, and IFN-γ.

### The in vivo effect of PD-L1-SARS peptide vaccination for tumor prevention on the growth and liver metastasis of B16F10 melanoma cells

**Fig. 2:**
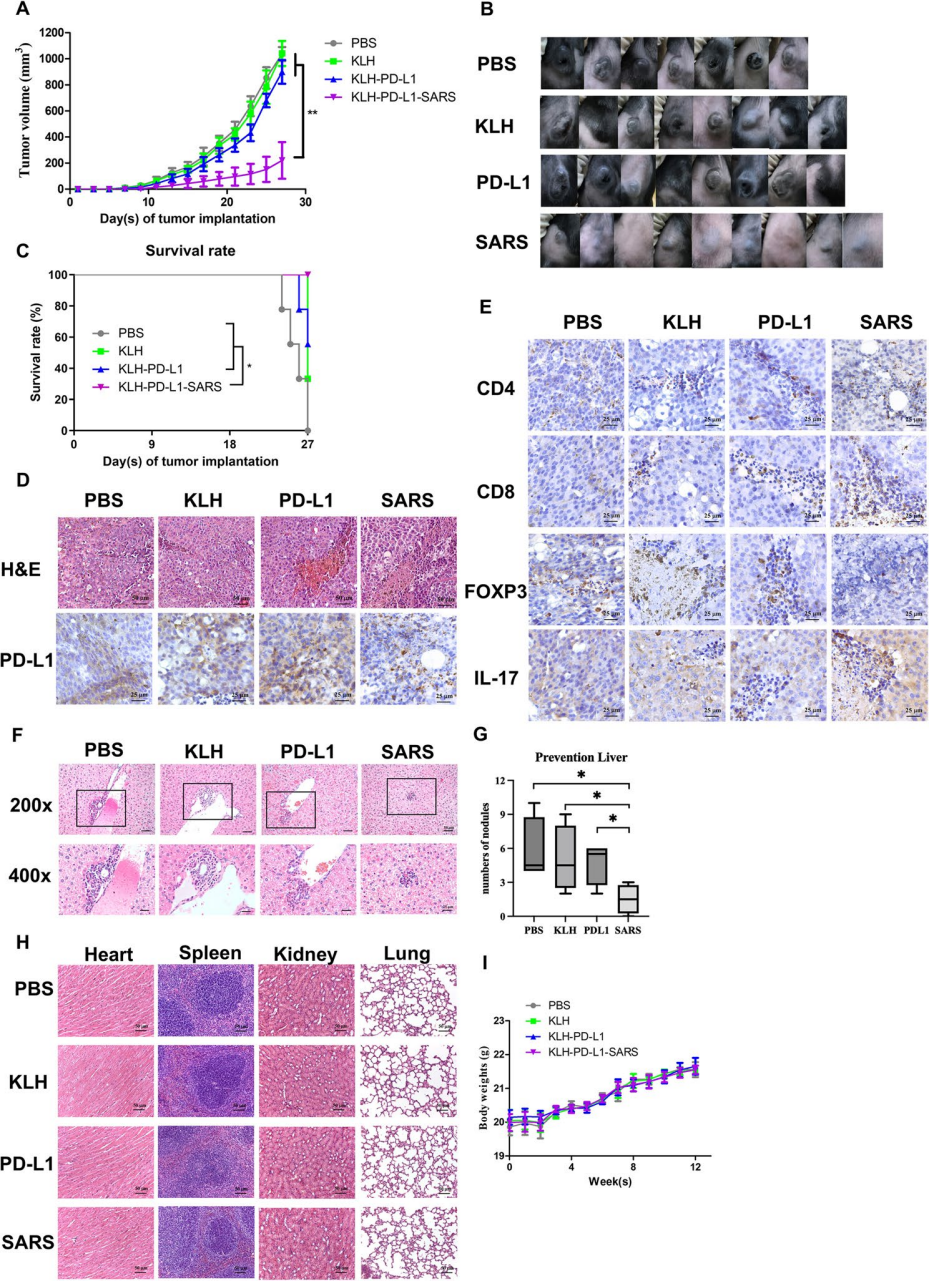
**The tumor prevention efficacy of the PD-L1-SARS peptide vaccine.** **A** The KLH-PD-L1-SARS peptide vaccine inhibited tumor growth more effectively than the KLH-PD-L1 peptide. A significant difference was indicated by ** (*P* < 0.01). Data were expressed as the mean ± SD (*n* = 7 ~ 9). **B** Representative image of the tumors in the mice on the 17th day. PD-L1 was indicated KLH-PD-L1, and SARS was KLH-PD-L1-SARS. **C** Mouse survival was monitored daily. The mice were sacrificed when the tumors exceeded 1,000 mm^3^ in size. All mice of KLH-PD-L1-SARS group survived on the 27th day. **D** Representative H&E staining image of a tumor. PD-L1 expression in the tumors was detected by immunohistochemical (IHC) staining, and representative images were obtained from the different treatment groups. The tumor was severely damaged, and PD-L1 expression was decreased in the KLH-PD-L1-SARS group. **E** IHC staining of CD4, CD8, FOXP3, and IL-17 was performed to analyze their expression, and representative images were obtained from the different treatment groups. The tumors in the KLH-PD-L1-SARS group presented higher CD4, CD8, and IL-17 expression but lower FOXP3 expression. **F** Representative H&E staining images of the liver. The square frame in the picture (200x) represented the melanoma colonies. The size of the colonies was smaller in the KLH-PD-L1-SARS group than in the other groups. **G** The numbers of melanoma nodules were calculated and showed as boxplots. KLH-PD-L1-SARS efficiently decreased the number of melanoma nodules in the liver. A significant difference was indicated by * (*P* < 0.05). Data were expressed as the mean ± SD. **H** Representative H&E staining images of the heart, spleen, kidney, and lung. There was no significant damage to the organs between the groups. **I** The body weights of the mice were not significantly different among the groups

To validate the efficacy of the peptide vaccine for tumor prevention, Fig. 2A showed that the KLH-PD-L1-SARS vaccination significantly inhibited tumor growth compared with the other groups. Interestingly, two mice in the KLH-PD-L1-SARS vaccination group were tumor-free on the 30th day after tumor implantation (Fig. 2B). On the 27th day, the mice in the PBS group were all humanely sacrificed. The survival rate was 60% for the KLH-PD-L1 group and only 30% for the KLH group; however, all the KLH-PD-L1-SARS-vaccinated mice survived (Fig. 2C). For transparency, a KLH-SARS-only arm was not carried forward into this confirmatory experiment because pilot studies showed no measurable benefit under identical conditions (Fig. S5); in line with 3R (Reduction/Refinement), animals were concentrated on the antigen-matched comparison.

In the KLH-PD-L1-SARS group, the H&E stain images revealed that the tumor tissues were significantly more damaged than those in the other treatments were (Fig. 2D). The IHC results showed that the expression of the PD-L1 antigen was lower in the KLH-PD-L1-SARS group than in the other groups. Additionally, the IHC results indicated an increase in CD4, CD8, and IL-17 expression in the tumor area of the mice that received the KLH-PD-L1-SARS vaccine (Fig. 2E). In contrast, the presence of FOXP3 molecules was lower in the KLH-PD-L1-SARS group than in the PBS, KLH, and KLH-PD-L1 groups (Fig. 2E). These findings suggested that KLH-PD-L1-SARS treatment promoted the infiltration of CD4^+^ T, CD8^+^ T, and Th17 cells while suppressing the infiltration of Treg cells into the tumor area.

In addition, the impact of KLH-PD-L1-SARS vaccination on distant metastasis was assessed, and the result indicated a significant reduction in the sizes and numbers of metastatic colonies in the liver (Fig. 2F and G). Figure 2H demonstrated that there were no visible pathological differences in the vital organs among all the treatment groups, and the body weights of the mice receiving the KLH-PD-L1-SARS vaccine were not significantly different from those of mice in the other groups (Fig. 2I). These results suggested that KLH-PD-L1-SARS vaccination did not induce severe or significant side effects.

Furthermore, healthy and non-tumor-implanted C57BL/6J mice were vaccinated once every two weeks until the 30th week. Figure S2A showed that the body weights of the mice still steadily increased under high anti-PD-L1 antibody titers during a long period of vaccination. Finally, the pathological images of the organs were examined, and they were not visibly damaged in any of the groups (Fig. S2B). Therefore, the abovementioned results indicated that the KLH-PD-L1-SARS vaccine exhibited effective prevention activity and safety in mice.

### The therapeutic effect of PD-L1-SARS peptide vaccination on the inhibition of B16F10 tumor growth and liver metastasis

**Fig. 3:**
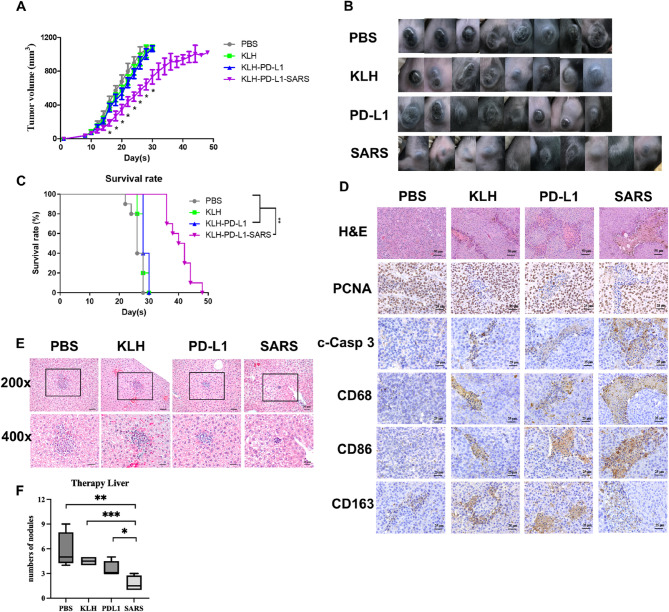
**The effects of in situ treatments on B16F10 tumor growth in vivo.** **A** The therapeutic effects of the PD-L1-SARS peptide vaccine on mice bearing B16F10 tumors. A significant difference compared with the KLH-PD-L1 group was indicated by * (*P* < 0.05). Data were expressed as the mean ± SD (*n* = 8 or 10). **B** Representative image of the tumors in the mice on the 17th day. **C** Mouse survival was monitored every two days, and the mice were sacrificed when the tumors exceeded 1,000 mm^3^ in size. **D** Representative H&E staining images of tumors and immunohistochemical staining of PCNA, cleaved caspase 3 (c-Casp 3), CD68, CD86, and CD163 were performed to analyze their expression in the mice in the different treatment groups. Representative images of the different treatment groups were shown. In the KLH-PD-L1-SARS group, the expression levels of c-Casp 3, CD68 and CD86 were higher than those in the other groups, but the expression levels of PCNA and CD163 were lower. **E** Representative H&E staining images of the liver. The square frame in the picture (200x) represented the melanoma colonies. The size of the colonies was smaller in the KLH-PD-L1-SARS group than in the other groups. Additional representative liver sections from multiple animals per group are provided in Supplementary Fig. S4. **F** The numbers of melanoma nodules were calculated and shown as boxplots. KLH-PD-L1-SARS efficiently decreased the number of melanoma nodules in the liver. A significant difference was indicated by * (*P* < 0.05), ** (*P* < 0.01), and *** (*P* < 0.001). Data were expressed as the mean ± SD

The therapeutic effects of KLH-PD-L1-SARS peptide vaccination on tumor growth and metastasis were subsequently evaluated via in situ injection. When the average volume of B16F10 melanoma in mice reached 30 mm^3^, different treatments were injected into the tumors to validate their activities in suppressing tumor growth. Figure 3A showed that B16F10 tumor growth did not significantly differ among the groups treated with PBS, KLH, and KLH-PD-L1 (Fig. 3A and B). However, compared with other treatments, KLH-PD-L1-SARS peptide treatment significantly suppressed tumor growth. Interestingly, it also efficiently prolonged survival (Fig. 3C).

H&E stain images showed that the in situ KLH-PD-L1-SARS caused more tumor cell death and bleeding in the tumor tissue compared with the KLH and KLH-PD-L1 groups (Fig. 3D). Additionally, KLH-PD-L1-SARS reduced the expression of PCNA in tumor cells, indicating a decrease in tumor cell proliferation. Compared with KLH and KLH-PD-L1, KLH-PD-L1 also had greater ability to activate caspase 3, leading to cell apoptosis (Fig. 3D).

Compared with other treatments, KLH-PD-L1-SARS treatment increased the levels of CD68 and CD86. However, it reduced the expression of CD163 antigens in tumor areas, indicating increased macrophage infiltration into the tumor area and differentiation into the M1 subtype of macrophages (Fig. 3D). Interestingly, the in situ therapeutic effect of KLH-PD-L1-SARS on distant metastasis to the liver revealed that the sizes and numbers of metastatic colonies were markedly lower than those in the other groups (Fig. 3E and F, and S4).

### Anti-PD-L1 therapeutic antibody could more efficiently suppress tumor growth with the assistance of the PD-L1-SARS peptide vaccination

**Fig. 4:**
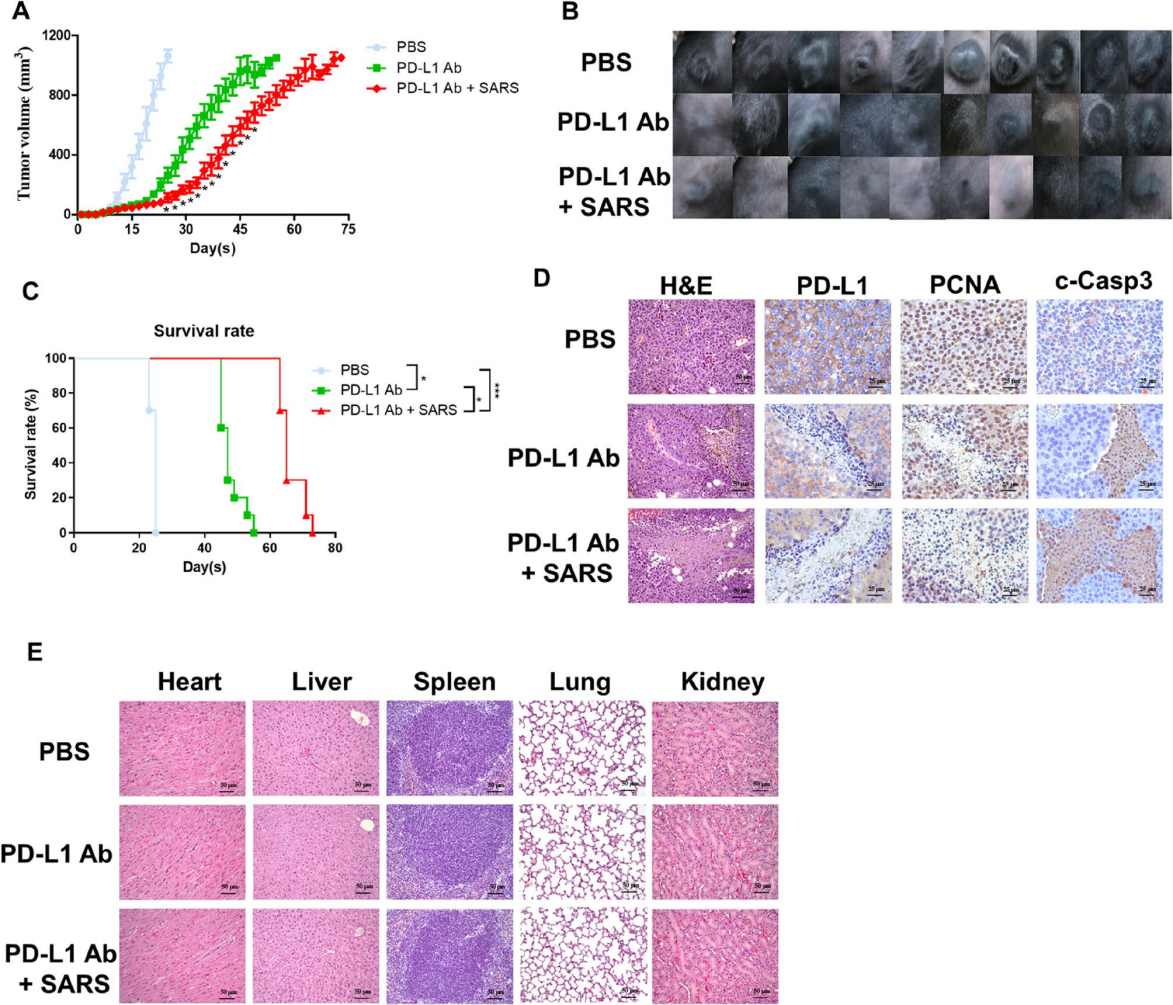
**The effects of combination therapy on B16F10 tumor growth in vivo.** **A** The growth inhibition effect of the PD-L1-SARS peptide vaccine and anti-PD-L1 antibody on B16F10 tumors was more significant than that of the anti-PD-L1 antibody alone. A significant difference compared to the PD-L1 antibody group was indicated by * (*P* < 0.05). Data were expressed as the mean ± SD (*n* = 10). **B** Representative image of the tumor on mice on the 25th day. **C** Mouse survival was monitored every two days, and the mice were sacrificed when the tumors exceeded 1,000 mm^3^ in size. Compared with single therapy, combined therapy prolonged mouse survival time. **D** Representative H&E staining image of a tumor. The expression of PD-L1, PCNA, and c-Casp3 in the tumors was determined by IHC, and representative images of the different treatment groups were obtained. Compared with single therapy, combined therapy efficiently increased the expression of c-Casp 3 but decreased PD-L1 and PCNA expression and caused damage to tumors. **E** Representative H&E staining images of the heart, liver, spleen, lung, and kidney. There was no significant damage to the organs between the groups

The anti-PD-L1 therapeutic antibodies as immune checkpoint inhibitors (ICIs) have been applied in human melanoma clinical therapy. Notably, the PD-L1-SARS peptide vaccine couldn’t produce antibodies recognized against PD-L1 antigens of the tumor surface, determined its adjuvant effect when combined with such ICIs therapy. The result indicated that treatment with the anti-PD-L1 antibody alone indeed suppressed tumor growth; however, tumor growth inhibition was further enhanced by the combination of this antibody with in situ injection of the PD-L1-SARS peptide vaccine (Fig. 4A and B). The survival rate of the combination therapy group was greater than that of the single therapy group (Fig. 4C).

Moreover, H&E stain images showed that the combination therapy caused more severe damage to the tumor tissue compared with the anti-PD-L1 antibody therapy (Fig. 4D). Compared with anti-PD-L1 antibody treatment, the combination therapy reduced the number of PD-L1-expressing melanoma cells more effectively. This could be attributed to the combination therapy’s more efficient reduction of cell proliferation (as indicated by PCNA expression) and promotion of cell apoptosis (as indicated by c-Casp3 expression) (Fig. 4D). Figure 4E indicated that no significant damage was observed in any of the normal organ tissues, including the heart, liver, spleen, lung, and kidney, for either of the treatments.

### SARS peptides stimulate antitumor immunity through the induction of IL-17 and IFN-g

**Fig. 5:**
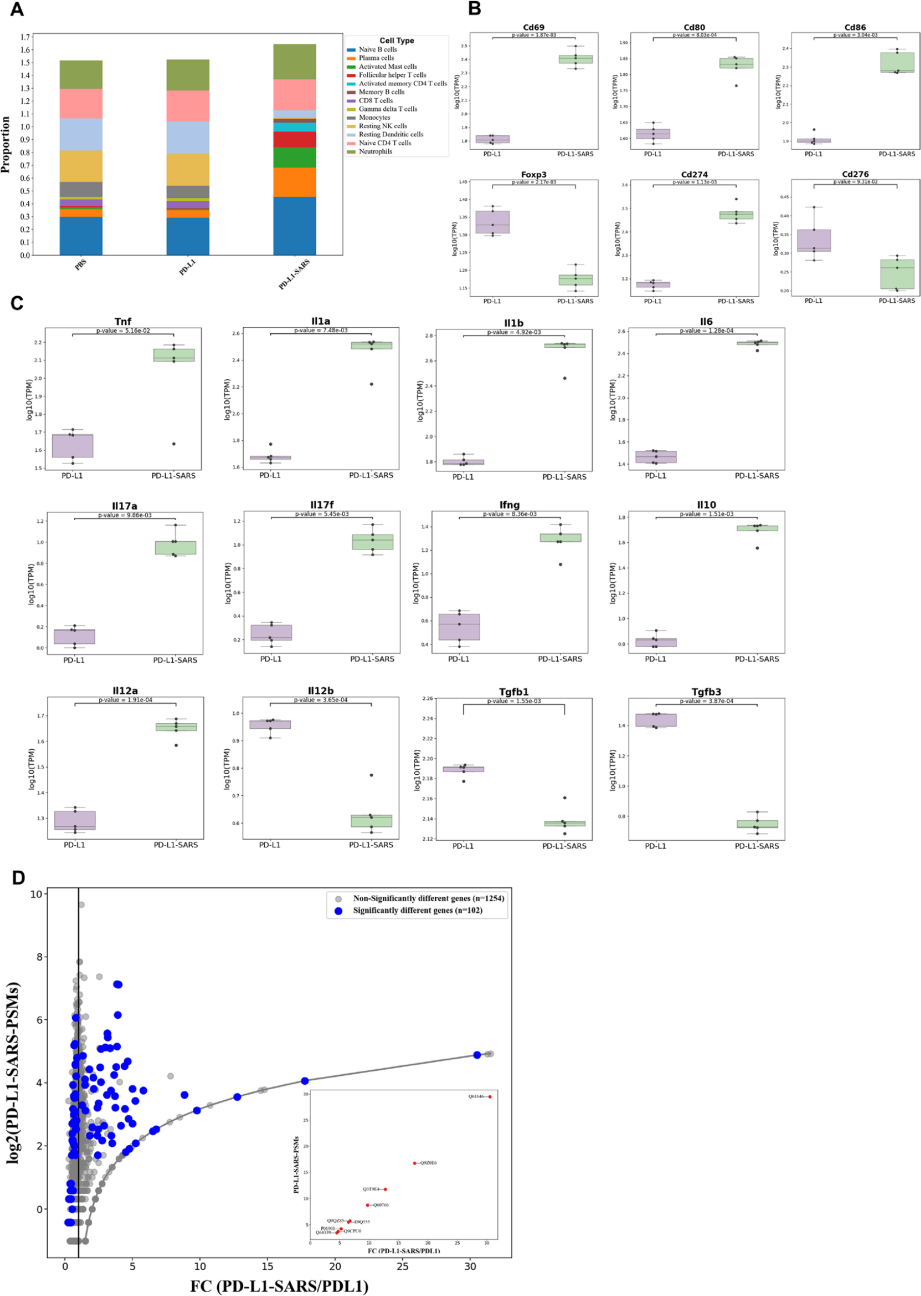
**PD-L1-SARS peptide enhances immune activation and antitumor response through differential gene expression.** A total of 851 differentially expressed genes (DEGs), identified by comparing the PD-L1 peptide group with the PD-L1-SARS peptide group, were further analyzed to investigate the abundance of associated cell types and the expression levels of CD markers. **A** The proportions of each cell type were calculated by CIBERSORTx. The proportions of naïve B-cells, plasma cells, activated mast cells, follicular Th cells, activated memory CD4^+^ T cells, and memory B-cells were increased in the PD-L1-SARS group. **B** The expression levels of the immune activation markers CD69, CD80, and CD86 on immune cells were increased, whereas those of the immunosuppressive marker FOXP3 were decreased. However, CD274 expression was increased. **C** Among the DEGs, key cytokines involved in inflammatory responses and other immune cytokines, including IL-12, IFN-γ, IL-17, IL-10, and TGF-β, were highlighted. Compared with treatment with the PD-L1 peptide, treatment with the PD-L1-SARS peptide significantly enhanced immune responses, suggesting that the PD-L1-SARS peptide had the potential to stimulate a more robust antitumor immune response. **D** The plot displayed the log2-transformed peptide spectrum matches (PSMs) values on the y-axis against the fold change (FC) values on the x-axis. The blue dots represented significantly different genes (*n* = 102), whereas the gray dots indicated non-significantly different genes (*n* = 1254). The alongside inset image showed that the selected genes (red spots) were significantly inducible, and that changes in expression were linear (Equation: y = x − 1). The numbers by the red spots were accession numbers in the UniPort database, and the functions of these nine proteins were shown in Table S3

CIBERSORTx analysis of the NGS data revealed changes in immune cell populations, as shown in Fig. 5A. Compared with the PD-L1 peptide, the PD-L1-SARS peptide significantly increased the proportions of naïve B-cells, plasma cells, activated mast cells, follicular Th cells, activated memory CD4^+^ T cells, and memory B-cells in the assay. Conversely, compared with the PD-L1 peptide, the PD-L1-SARS peptide reduced the proportions of CD8^+^ T cells, monocytes, resting NK cells, and resting dendritic cells. These results indicated that administering PD-L1-SARS peptides stimulated B- and T-cell activity and differentiation.

As expected, the immune markers of active T cells, B cells, DCs, and macrophages, such as CD69, CD80, and CD86, were upregulated in the PD-L1-SARS group compared with the PD-L1 group. Conversely, the expression of the immunosuppressive marker FOXP3 was decreased in the PD-L1-SARS group, whereas that of CD276 was not significantly different, and that of CD274 was increased (Fig. 5B). These results suggested that the PD-L1-SARS peptide could directly activate immune cells and attenuate immunosuppression.

Furthermore, the PD-L1-SARS peptide effectively increased the levels of proinflammatory cytokines such as TNF-α, IL-1α, IL-1β, and IL-6 but not IL-13 (Fig. 5C and S3). Additionally, the expression of the Th1 activating-cytokine IL-12, namely IL-12α, was elevated in the PD-L1-SARS group, whereas the expression of the IL-12β gene was decreased (Fig. 5C). Subsequent monitoring of the IL-17 family revealed increased expression of IL-17a and IL-17f, whereas the expression of IL-17b, IL-17c, and IL-17d was not significantly different (Fig. 5C and S3). Moreover, the levels of type II IFN, IFN-γ, were also increased, while the levels of type I IFNs (IFN-α and IFN-β) and other IFN family members were not significantly different (Fig. 5C and S3). In contrast, the PD-L1-SARS peptide significantly decreased the levels of immunosuppressive cytokines, including TGF-β1 and TGF-β3, but increased the level of IL-10 (Fig. 5C). Therefore, the results indicated that the PD-L1-SARS peptide efficiently activated the antitumor immune response by increasing the levels of proinflammatory cytokines, IL-17, and IFN-γ and decreasing the levels of immunosuppressive cytokines, such as TGF-β.

LC‒MS/MS identified significant protein differences between PD-L1-SARS and PD-L1 peptide treatments in the tumor microenvironment (TME). The peptide spectrum matches (PSMs) and fold changes (FCs) of 102 significant proteins and 1254 insignificant proteins were displayed in Fig. 5D. We identified nine proteins with high FC values and significant expressions. These proteins followed the formula y = x − 1. On the basis of our calculation via the formula FC = (PSMs of the specific protein in the PD-L1-SARS group + 1)/(PSMs of the specific protein in the PD-L group + 1), the nine proteins were not expressed in the PD-L1 peptide group but were highly expressed in the PD-L1-SARS peptide group.

The names and accession numbers of the nine proteins were as follows (also listed in Table S3): haptoglobin (Q61646), IFN-induced guanylate-binding protein 2 (Q9Z0E6), IFN-γ-inducible GTPase ifggb6 protein (Q3T9E4), IFN-inducible GTPase 3 (Q60766), IFN-inducible GTPase 1 (Q9QZ85), E3 ubiquitin-protein ligase (E9Q555), H-2 class I MHC (IFN-induced protein, P01901), lactoylglutathione lyase (Q9CPU0), and IFN-induced 15 kDa protein (Q64339). According to the published literature, eight out of the nine proteins were involved in the IFN-γ-related pathway (Table S3). In addition, lactoylglutathione lyase catalyzed the formation of S-lactoylglutathione, which was involved in the transcription of the proinflammatory cytokine TNF-α. These results indicated that the PD-L1-SARS peptide could induce the expression of IFN-γ and TNF-α and to promote their activities in immune activation and inflammation in the TME.

### Exploratory in silico prioritization of candidate receptors potentially linked to IL-17/IFN-γ induction

**Fig. 6:**
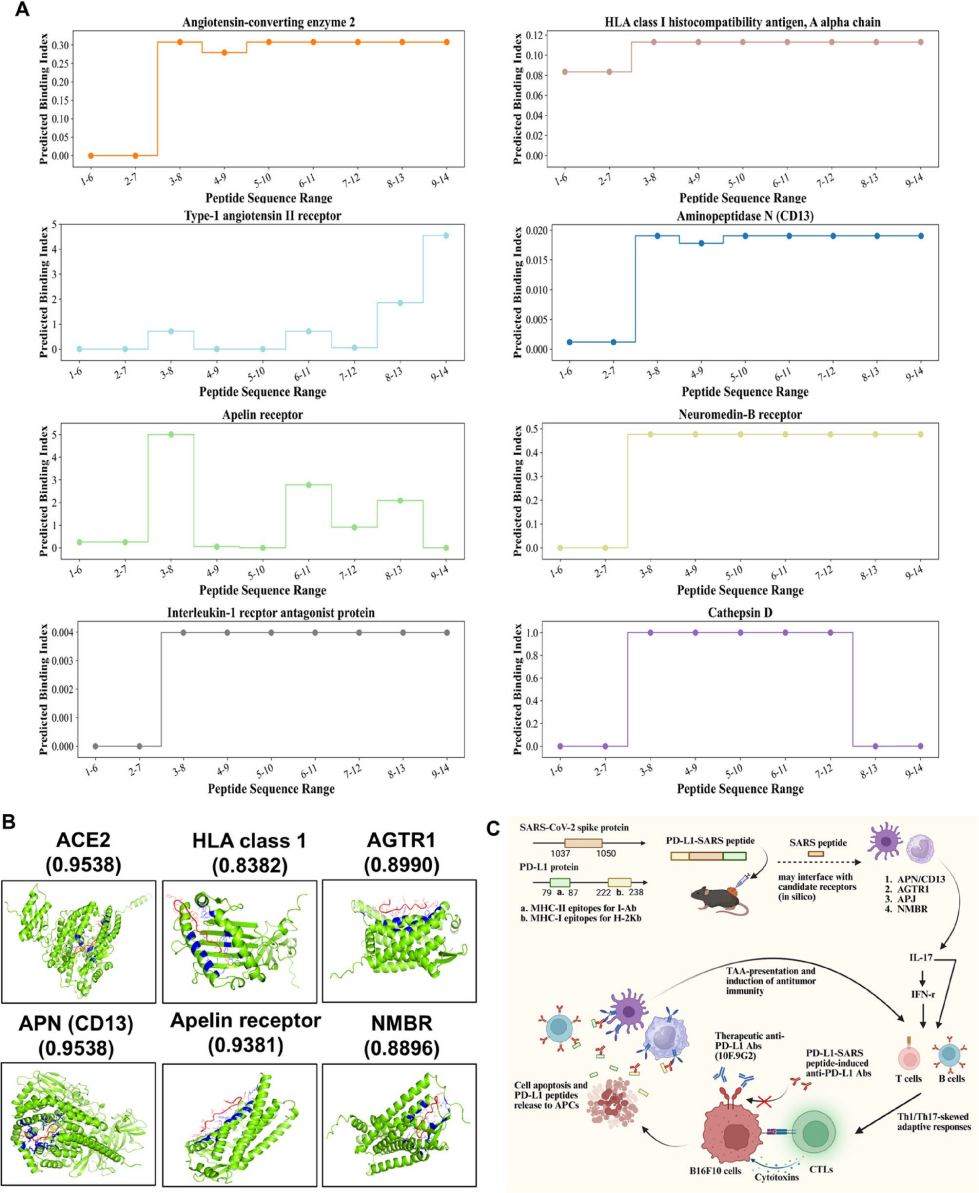
**Hypothesis-generating in silico screen prioritizes candidate receptors potentially engaged by the SARS-derived peptide.** This analysis is exploratory and does not constitute evidence of physical binding or causality; experimental validation is required. **A** The 14-aa SARS peptide was segmented into overlapping 6-aa subfragments and subjected to peptidomimetic reverse docking. The y-axis shows the Predicted binding index (unitless), a display of BindingDB literature affinities for small-molecule mimetics of 6-aa subfragments (reported as 1/Kd in nM⁻¹ or inverted from Kd/Ki/IC₅₀); larger values indicate stronger predicted interaction propensity (see Table S4). This index is computational and not an experimental affinity of the peptide. The panel lists eight putative targets recovered by the screen; ACE2 and MHC-I appear as face-validity/internal controls given prior literature, supporting the plausibility—but not validating—the computation. **B** Representative docking poses of the full-length peptide against six prioritized proteins are shown (peptide in red; receptor in green; putative contact residues/sites in blue), together with the corresponding confidence scores. Based on these scores, cathepsin D and interleukin-1 receptor antagonist were de-prioritized. Abbreviations: AGTR1, type-1 angiotensin II receptor; APN, aminopeptidase N; NMBR, neuromedin-B receptor. **C** The PD-L1 peptide supplies antigenic epitopes for presentation on MHC-I/II, while the SARS-derived motif may provide adjuvant-like priming, potentially interfacing with candidate receptors (in silico)—APN/CD13, AGTR1, APJ, and NMBR—to amplify innate signals. This promotes Th1/Th17-skewed adaptive responses (↑IL-17, ↑IFN-γ; B-cell and T-cell activation), and CD8⁺ CTL cytotoxicity, shifting the TME from suppressive to activating. In the combination setting, vaccine-elicited PD-L1–specific antibodies and CTLs are complemented by therapeutic anti-PD-L1 mAb (10 F.9G2), which releases PD-1/PD-L1 inhibition on tumor cells; tumor cell killing further releases PD-L1 peptides that are captured by APCs, reinforcing the loop. Together, these coordinated effects provide a mechanistic rationale for the enhanced melanoma control observed in vivo

We computationally profiled the 14-amino-acid SARS-derived peptide by differentiating its sequence, predicting structure, and performing reverse docking to prioritize candidate host receptors. Figure 6A prioritizes eight candidate proteins predicted to interact with the overlapping 6-aa subfragments; the corresponding predicted binding index (unitless) are summarized in Table S4. These in silico scores are hypothesis-generating and do not represent experimental affinities. Among these, ACE2 (previously reported to bind SARS-CoV-2 spike) and MHC class I (predicted by IEDB to present the peptide) were recovered as internal/face-validity controls, supporting the plausibility—but not validating—the computational screen.

Figure 6B shows representative docking poses between the full peptide and the shortlisted proteins. Based on confidence scores, cathepsin D and interleukin-1 receptor antagonist were deprioritized from the initial set. Literature mapping (see Table S5) indicates that several candidate receptors are mechanistically connected to dendritic cell, macrophage, B-cell, or T-cell activation and have been associated with IL-17 and/or IFN-γ biology, providing contextual coherence with our in vitro/in vivo cytokine, IHC, and RNA-seq findings. As an initial, exploratory cross-check consistent with this framing, a plate-based binding ELISA using a biotinylated SARS-derived peptide and immobilized receptors (APJ, APN/CD13, AGTR1, NMBR) showed dose-dependent OD₄₅₀ signals, with APJ and APN generally higher than AGTR1/NMBR (Fig. S6). Because plate-coating assays are subject to orientation/avidity artifacts, these data are qualitative and do not establish affinity or physical binding; accordingly, Fig. 6 remains hypothesis-generating.

Figure 6C shows hypothesis-generating working model for how a PD-L1–SARS peptide may re-condition the TME and why it synergizes with anti-PD-L1. The PD-L1 peptide supplies antigenic epitopes for MHC-I/II presentation, whereas the SARS-derived motif may provide adjuvant-like priming. In silico docking nominates candidate receptor/pathway interfaces (e.g., APN/CD13, AGTR1, APJ, NMBR) that could link the motif to innate sensing. Together, the vaccine program may enhance antigen presentation and myeloid polarization (↑CD86/CD163), followed by Th1/Th17-skewed adaptive responses (↑IFN-γ, ↑IL-17), B-cell activation/anti–PD-L1 antibodies, and CD8⁺ CTL cytotoxicity, consistent with tumor control. In combination therapy, the therapeutic anti-PD-L1 mAb (10 F.9G2) releases PD-1/PD-L1 inhibition on T cells; CTL killing and antibody-mediated opsonization promote tumor-cell apoptosis and release of PD-L1 antigen, which is captured by APCs, reinforcing a positive feedback loop of antigen uptake, presentation, and effector activation.

## Discussion

This study is the first to demonstrate that a PD-L1-SARS fusion peptide, incorporating a SARS-CoV-2 spike fragment, can enhance the immunogenicity of a PD-L1 peptide and activate antitumor responses. In both prophylactic and therapeutic models, the vaccine significantly inhibited melanoma growth and metastasis. Combination with anti-PD-L1 antibodies further improved therapeutic outcomes. Mechanistic insights from RNA-seq, LC–MS/MS, and an in silico, hypothesis-generating docking screen suggest activation of macrophage and dendritic-cell programs, with increased IL-17 and IFN-γ signatures compatible with a Th1-biased response. In situ vaccination using this peptide represents an effective strategy to induce robust tumor-specific immunity [30].

The SARS-CoV-2 spike protein has been shown to induce cytokine expression through TLR2 [14] and TLR4 [15], promoting the release of inflammatory mediators including IFN-γ [31]. Consistently, Fig. 1E and F illustrate that the PD-L1-SARS peptide enhances the secretion of proinflammatory cytokines and chemokines. Consistent with the behavior of many peptide-based adjuvants, we observed a transient elevation of innate pro-inflammatory mediators (e.g., TNF-α, IL-1, IL-6), which likely reflects early adjuvant priming rather than a sustained effector state. By contrast, the dominant and durable adaptive signature in our model is IFN-γ/IL-17-centric and temporally coincides with antitumor activity, as supported by cellular and tissue readouts (e.g., increased IFN-γ responses, enrichment of IL-17–related signals, CD8⁺ infiltration with reduced Treg markers, and macrophage polarization consistent with an M1-like phenotype). While IL-4/IL-13 increases were detectable at certain time points, their presence does not necessarily imply a net immunosuppressive program in this context; rather, the integrated immune milieu is compatible with an effective Th1/Th17-biased antitumor response. Additionally, while IL-4 ELISpot also increased (Fig. 1D), this likely reflects early helper signals and B-cell support that can accompany vaccine-induced priming. Importantly, the net immune contexture in tumors—CD8⁺ infiltration, FOXP3 reduction, M1-type macrophage increasing, and IFN-γ–responsive protein enrichment—also indicates a Th1/Th17-dominant state that coincides with tumor control. We therefore summarize both absolute SFCs and a Th1/Th2 balance index (IFN-γ/IL-4) to emphasize the overall bias rather than any single cytokine in isolation. Furthermore, bioinformatics analyses further suggest that the SARS peptide interacts with multiple immune-related proteins involved in IL-17 and IFN-γ signaling (Figs. 5 and 6, Table S3–S5), supporting its role in driving IFN-γ-dependent antitumor immunity.

PD-L1-SARS vaccination activates both Th1 and Th2 responses and induces antibodies that selectively recognize degraded PD-L1 peptides from apoptotic tumor cells, without targeting membrane-bound PD-L1 or interfering with ICIs. These antibodies form immunocomplexes that are internalized by B cells, dendritic cells, and macrophages via Fc receptors, leading to T-cell activation and establishing a positive feedback loop that amplifies antitumor immunity. Therefore, we propose that in situ PD-L1-SARS peptide administration promotes the release of IL-17 and IFN-γ from macrophages and dendritic cells, enhancing the recruitment and activation of adaptive immune cells. By converting “cold” tumors into “hot” ones, this strategy improves ICI responsiveness, mitigates immune suppression and resistance, and facilitates sustained infiltration of T cells and macrophages for effective tumor clearance. These findings underscore the potential of SARS-CoV-2 spike-derived peptides as novel adjuvants in cancer immunotherapy [32].

Based on our in silico, hypothesis-generating screen (Fig. 6 and Table S5), the SARS-CoV-2 spike–derived peptide was predicted to interact with candidate receptors including ACE2, HLA class I, type-1 angiotensin II receptor (AGTR1), aminopeptidase N (APN/CD13), apelin receptor (APJ), and neuromedin-B receptor (NMBR). ACE2 is crucial in the renin-angiotensin system and serves as the entry receptor for SARS-CoV-2. Dysregulation of ACE2 may lead to an imbalance in angiotensin II levels, which promotes inflammation. Elevated angiotensin II could activate proinflammatory pathways, leading to increased IL-17 production by stimulating Th17 cell differentiation [33]. AGTR1 mediates most of the effects of angiotensin II, including vasoconstriction and proinflammatory responses. The activation of AGTR1 by angiotensin II enhances the production of reactive oxygen species and activates NF-κB, a transcription factor that promotes IL-17 expression [34]. APN, also known as CD13, is involved in immune regulation and inflammatory responses. APN modulates T-cell activation and cytokine secretion [35]. Its activation can influence Th17 cell responses, potentially leading to increased IL-17 expression through the modulation of signaling pathways such as the MAPK and NF-κB pathways [36]. APJ interacts with the peptide apelin and is involved in cardiovascular functions and fluid homeostasis. The activation of APJ has been shown to modulate immune responses. It can inhibit angiotensin II-induced effects, which may indirectly reduce IL-17 expression by counteracting AGTR1-mediated proinflammatory pathways [33]. NMBR is a G-protein coupled receptor that binds neuromedin-B, a peptide involved in various physiological processes. The activation of NMBR can influence proinflammatory cytokine release in arthritis or asthma models, potentially by inducing IL-17 expression [37, 38]. According to the literature, the interplay between these receptors and IL-17 involves complex signaling mechanisms that contribute to inflammation. Dysregulation or activation of ACE2, AGTR1, APN, APJ, and NMBR can modulate IL-17 expression by affecting Th17 cell differentiation and proinflammatory signaling pathways. Moreover, IL-17 enhances immunity through the induction of IFN-γ [39, 40]. IFN-g is a robust immune activator. Therefore, multiple pathways of IFN-γ production could be activated simultaneously. This may explain the putative mechanism by which the SARS fragment in the PD-L1-SARS peptide contributes to its adjuvant effect.

Figure 5D and Table S3 reveal nine proteins increased in the TME following PD-L1-SARS, eight of which annotate to IFN-γ–linked pathways, supporting a shift toward an IFN-γ–centric program. Among them, lactoylglutathione lyase has been reported to generate S-lactoylglutathione and thereby activate the NF-κB pathway, leading to TNF-α production [41]. In our dataset, PD-L1-SARS induced IFN-γ and TNF-α (Fig. 1E). Notably, IFN-γ and TNF-α can synergize to trigger caspase-8–dependent tumor cell death [42], which is compatible with the increased cytotoxicity observed in our assays (Figs. 1C and 3D). Besides, IFN-γ could drive Treg fragility to promote antitumor immunity [43], and such a reduction in Tregs in the TME was shown in Fig. 2E. The results of this study suggested that the PD-L1-SARS peptide could trigger IL-17 production (Fig. 2E), induce IFN-γ production [39, 40], and synergically promote inflammatory cytokine production [44]. Finally, these cytokines lead to the enhancement of Th1 cells and a decrease in Treg cells, which strengthen the antitumor immunity. Furthermore, the SARS peptide increased CD68 and CD86 expression while decreasing CD163 expression, indicating that macrophages polarization was shifted toward the M1 type (Fig. 3D). Together with the efficacy readouts, these observations support a transition from an immune-suppressive to an immune-activating TME under PD-L1-SARS. In addition to macrophage and T-cell changes, the B-cell compartment expanded (including plasma and memory B cells; Fig. 5A), consistent with PD-L1–specific humoral responses and enhanced antigen handling within tumors. Mechanistically, B cells can present peptide–MHC II to CD4⁺ T cells, indirectly facilitating CD8⁺ priming; antibody-opsonized antigen can be captured by professional APCs via Fcγ receptors, improving uptake and cross-presentation; and B-cell aggregation may favor tertiary lymphoid structure (TLS)–like microenvironments that coordinate local T–B interactions. In line with these roles, PD-L1-SARS is accompanied by a Th1/Th17-leaning immune contexture (IFN-γ and IL-17 signals; CD8⁺ infiltration↑, FOXP3⁺ Tregs↓, M1-type macrophage polarization) and IFN-γ–linked proteomic enrichment. Finally, the observed mast-cell increase and monocyte decrease may reflect adjuvant-driven myeloid remodeling; importantly, the net signature across lymphoid and myeloid compartments remains Th1/Th17-leaning and coincides with tumor control.

Notably, IFN-γ acts as a double-edged sword in tumor growth. The expression of IFN-γ induces PD-L1 (CD274) expression, as shown in Fig. 5B [45]. However, administering the PD-L1-SARS peptide stimulated IFN-γ production while simultaneously reducing PD-L1 expression in the tumor region (Fig. 2D). We propose that the PD-L1-SARS peptide enhances the cytotoxic Th1 cell response, destroying tumor cells that express PD-L1. Consequently, melanoma cells that overexpress PD-L1 are particularly susceptible to being targeted and eliminated.

This study utilized a combination of three methods to investigate how SARS peptides activate antitumor immune responses. We applied an incremental, fragment-based molecular docking strategy in which the SARS-derived peptide was partitioned into overlapping subfragments. This in silico screen was used to prioritize putative receptor pockets and to nominate targets whose reported biology aligns with innate/adaptive immune pathways. These analyses are hypothesis-generating and do not establish physical binding or immunological activity. Furthermore, RNA-seq analysis showed that the SARS peptide increased the expression of immune activation-related markers and cytokines and decreased the expression of immunotolerant molecules and inhibitory cytokines. Furthermore, CIBERSORTx indicated that the proportions of various B- and T-cell populations were elevated in the PD-L1-SARS group. These results showed that the PD-L1-SARS peptide could increase the activities of immune cells and reduce the levels of immunosuppressive factors to switch cold tumors to hot tumors. LC‒MS/MS data analysis also proved that the SARS peptide induced the IFN-γ pathway for the induction of antitumor immune responses and suppression of malignancy in the TME.

Traditional carriers (e.g., KLH) can present peptide antigens but often favor humoral responses and, as seen with KLH-PD-L1 in this study, may insufficiently activate intratumoral cellular immunity. Classical adjuvants may skew toward Th2 (e.g., alum) or depend on strong TLR agonism that requires careful formulation to mitigate systemic reactogenicity. By contrast, our PD-L1-SARS design embeds an adjuvant-like motif within the vaccine and is delivered in situ, aiming to pair antigenic specificity with local innate priming. Consistent with this intent, the PD-L1-SARS arm shows a Th1/Th17-leaning program, CD8⁺ infiltration, FOXP3⁺ Treg reduction, and M1-leaning macrophage polarization, together with IFN-γ-linked protein enrichment in the TME—features consistent with re-conditioning a suppressive microenvironment. These properties may help address a key limitation of PD-L1 peptide vaccines given with carrier alone, which struggle to convert “cold” tumors. Compared with checkpoint antibodies, which primarily release inhibitory signaling but do not provide antigen, our approach supplies defined antigenic epitopes and may prime de novo immunity in antigen-poor or “cold” settings; the combination benefit observed in our models supports a complementary (not competing) role. Practically, a defined peptide construct offers modularity and may reduce off-target exposure with in situ dosing. We note that receptor-level mechanisms remain hypothesis-generating and safety/dose optimization will require future, dedicated studies.

## Conclusion

We demonstrated that the SARS peptide efficiently assisted anti-PD-L1 immune responses against B16F10 melanoma cells both in vitro and in vivo. Unlike small-molecule/chemotype approaches that target PD-L1 or TMDs directly, our vaccine supplies antigen and locally conditions the TME to support CTL function; as such, it can complement checkpoint blockade. We propose that the role of the SARS peptide in the antitumor mechanism may indicate its value as a new therapeutic vaccine for tumor immunotherapy.

## Supplementary Information

Supplementary Material 1.

## Data Availability

All data generated or analyzed during this study are available from the corresponding author upon reasonable request. In addition, all supporting next-generation sequencing (NGS) data have been deposited in the Gene Expression Omnibus (GEO) database under accession number PRJNA1007364.
