# Supplementary material for: A SARS-CoV-2 spike-derived adjuvant peptide boosts IL-17/IFN-γ immunity and improves anti-PD-L1 therapy against melanoma
Source: Mol Med. 2025 Dec 27;31:338. doi: 10.1186/s10020-025-01384-2 (PMC12743402; doi:10.1186/s10020-025-01384-2)
Supplement: Supplementary file 1 — Supplementary Material 1. [file 10020_2025_1384_MOESM1_ESM.docx]

**Supplementary material**

|  |  |  |  |
| --- | --- | --- | --- |
| **Peptides** | **Description** | **Amino acid sequences of synthesized peptides** | **Length** |
| PD-L1 | PDL1^(222-238)^-PDL1^(79-87)^ | H_2_N-^222^ELIIPELPATHPPQNRT^238^-^79^SNFRGRASL^87^-CONH_2_ | 26 mer |
| PD-L1-SARS | PDL1^(222-238)^-SARS spike protein^(1037-1050)^-PDL1^(79-87)^ | H_2_N-^222^ELIIPELPATHPPQNRT^238^-^1037^AAPHGVVFLHVTYV^1050^-^79^SNFRGRASL^87^-CONH_2_ | 40 mer |
|  |  |  |  |

**Table S1. The amino acid sequences of the PD-L1 and PD-L1-SARS peptides.** The table listed the amino acid sequences and composition information of the two fusion peptides.

| **The specific antibodies were used in IHC experiment.** | | | | |
| --- | --- | --- | --- | --- |
| **Antibody** | **Companies purchased from** | **Dilution** | **Clone** | **Catalogue NO.** |
| PCNA | Cell Signaling Technology | 1:6000 | D3H8P | #13110 |
| cleaved caspase 3 | Cell Signaling Technology | 1:2000 | 5A1E | #9664 |
| PD-L1 | Arigo | 1:500 | Polyclonal | ARG55930 |
| IL-17 | Arigo | 1:400 | Polyclonal | ARG55256 |
| CD4 | Abcam | 1:1000 | EPR19514 | ab183685 |
| CD8 | Cell Signaling Technology | 1:800 | D4W2Z | #98941 |
| CD68 | Abcam | 1:5000 | Polyclonal | ab125212 |
| CD86 | Cell Signaling Technology | 1:500 | E5W6H | #19589 |
| CD163 | Abcam | 1:500 | EPR19518 | ab182422 |
| FOXP3 | Arigo | 1:1000 | Polyclonal | ARG63507 |

**Table S2. Specific antibodies used in the IHC experiment.** The table included the antibody names (e.g., PCNA, cleaved caspase 3, PD-L1, and IL-17), sources (e.g., Cell Signaling Technology, Arigo), dilution ratios, clone types (e.g., D3H8P, polyclonal), and catalog numbers. This comprehensive information ensures reproducibility and reliability in experimental procedures.

| **Accession** | **PSMs** | **FC** | **Protein names** | **Related to immune responses** |
| --- | --- | --- | --- | --- |
| Q64339 | 3.5 | 4.5 | Interferon-induced 15 kDa protein | Belong to interferon-stimulated gene (ISG) |
| Q9CPU0 | 3.75 | 4.75 | Lactoylglutathione lyase | Related to TNF-α response [1]. |
| P01901 | 4.25 | 5.25 | H-2 class I MHC (IFN-induced protein) | Belong to ISG |
| E9Q555 | 5.5 | 6.5 | E3 ubiquitin-protein ligase | Modulated IFN-I response [2, 3]. |
| Q9QZ85 | 5.75 | 6.75 | Interferon-inducible GTPase 1 | Belong to ISG |
| Q60766 | 8.75 | 9.75 | Interferon-inducible GTPase 3 | Belong to ISG |
| Q3T9E4 | 11.75 | 12.75 | Interferon-gamma-inducible GTPase Ifggb6 protein | Belong to ISG |
| Q9Z0E6 | 16.75 | 17.75 | Interferon-induced guanylate-binding protein 2 | Belong to ISG |
| Q61646 | 29.5 | 30.5 | Haptoglobin | Stimulated IFN-β [4]. |

**Table S3. Summary of the significantly induced proteins.** The nine spots (proteins) identified from the comparison between the PD-L1-SARS and PD-L1 groups via LC‒MS/MS analysis were shown. Q9CPU0 was a lactoylglutathione lyase involved in the transcription of the proinflammatory cytokine TNF-α. E9Q555 was an E3 ubiquitin-protein ligase that modulated type I interferon (IFN-I) response. Q61646 was a haptoglobin that stimulated IFN-β via a TLR4-dependent mechanism. The other genes were interferon-stimulated genes (ISGs). PSMs: peptide spectrum matches; FC: fold change.

**Reference**

1. de Hemptinne, V., D. Rondas, M. Toepoel, and K. Vancompernolle, Phosphorylation on Thr-106 and NO-modification of glyoxalase I suppress the TNF-induced transcriptional activity of NF-kappaB. Mol Cell Biochem, 2009. 325(1-2): p. 169-78.

2. Nair, S., P. Bist, N. Dikshit, and M.N. Krishnan, Global functional profiling of human ubiquitome identifies E3 ubiquitin ligase DCST1 as a novel negative regulator of Type-I interferon signaling. Sci Rep, 2016. 6: p. 36179.

3. Liu, W., C. Yuan, B. Fu, J. Xie, W. Li, G. Zhang, et al., E3 ubiquitin ligase ANKIB1 attenuates antiviral immune responses by promoting K48-linked polyubiquitination of MAVS. Cell Rep, 2024. 43(9): p. 114687.

4. Kwon, J.O., W.J. Jin, B. Kim, H. Ha, H.H. Kim, and Z.H. Lee, Haptoglobin Acts as a TLR4 Ligand to Suppress Osteoclastogenesis via the TLR4-IFN-beta Axis. J Immunol, 2019. 202(12): p. 3359-3369.

| **Predicted binding index (unitless; higher = stronger)** | | | | | | | | | |
| --- | --- | --- | --- | --- | --- | --- | --- | --- | --- |
| **Protein name/ Peptide sequence** | **1-6** | **2-7** | **3-8** | **4-9** | **5-10** | **6-11** | **7-12** | **8-13** | **9-14** |
| Angiotensin-converting enzyme 2 | 0 | 0 | 0.3077 | 0.2793 | 0.3077 | 0.3077 | 0.3077 | 0.3077 | 0.3077 |
| HLA class I histocompatibility antigen | 0.0833 | 0.0833 | 0.113 | 0.113 | 0.113 | 0.113 | 0.113 | 0.113 | 0.113 |
| Type-1 angiotensin II receptor (AGTR1) | 0.0033 | 0.0033 | 0.7143 | 0.0025 | 0.0025 | 0.7143 | 0.0546 | 1.8519 | 4.5455 |
| Aminopeptidase N (CD13) | 0.0012 | 0.0012 | 0.019 | 0.0178 | 0.019 | 0.019 | 0.019 | 0.019 | 0.019 |
| Apelin receptor | 0.25 | 0.25 | 5 | 0.05 | 0 | 2.7778 | 0.9091 | 2.0833 | 0 |
| Neuromedin-B receptor (NMBR) | 0 | 0 | 0.004 | 0.004 | 0.004 | 0.004 | 0.004 | 0.004 | 0.004 |
| Interleukin-1 recptor antagonist protein | 0 | 0 | 0.4762 | 0.4762 | 0.4762 | 0.4762 | 0.4762 | 0.4762 | 0.4762 |
| Cathepsin D | 0 | 0 | 1 | 1 | 1 | 1 | 1 | 0 | 0.001 |

**Table S4. Predicted binding index (unitless) for candidate receptor–peptide subfragment pairs derived from peptidomimetic/BindingDB screening.** The table lists unitless predicted binding index from *in silico* docking for peptide sequences (columns, labeled by start–end positions, e.g., 1–6) against protein targets (rows). Higher index indicates a stronger predicted interaction. Predicted binding index values are derived from BindingDB literature affinities of small-molecule ligands topologically similar to 6-aa subfragments (reported as 1/Kd in nM⁻¹ or inverted from Kd/Ki/IC₅₀ in nM). Values are unitless and used descriptively to indicate predicted interaction propensity; they are not experimental affinities of the peptide and do not imply physical binding or causality.

| **Possible targets of SARS peptide** | **Immune responses associated with Macrophages, DCs and T cells. / IL-17 and IFN-γ expression.** |
| --- | --- |
| **ACE2** | Activation of DCs and Macrophages, and trigger proinflammation [5,6]. |
|  | Indirectly led to an increase in IL-17 production [7]. |
| **HLA class I** | Antigen presentation of DCs and Macrophages, and activation of T cells [8]. |
|  | It induced IFN-γ expression, and also was inducible-expressed by IFN-γ. |
| **Type-1 angiotensin II receptor (AGTR1)** | Differentiation of Th1 cells and M1 Macrophages [9-12]. |
|  | Promoted IL-17 expression [13]. |
| **Aminopeptidase N (APN/CD13)** | Antigen uptake and cross-presentation of DCs, activation of T cells and Macrophages [14, 15]. |
|  | Increased IL-17 expression [16, 17]. |
| **Apelin receptor (APJ)** | Accumulation of CD8 and CD4 T cells; M2 Macrophages polarization [18, 19]. |
|  | Indirectly reduced IL-17 expression [20]. |
| **Neuromedin-B receptor (NMBR)** | Recruitment of CD8 T cells and Macrophages [21]. |
|  | Potentially induced IL-17 expression [22, 23]. |

**Table S5. Literature mapping of docked candidate receptors to immune-cell pathways and IL-17/IFN-γ biology.** A molecular docking screen shortlisted candidate receptors potentially engaged by the SARS-derived peptide. This table contextualizes each candidate by summarizing reported links to immune-cell activation (e.g., dendritic cells, macrophages, B cells, T cells) and associations with IL-17 and/or IFN-γ from prior studies.

**Reference**

5. Labzin, L.I., K.Y. Chew, K. Eschke, X. Wang, T. Esposito, C.J. Stocks, et al., Macrophage ACE2 is necessary for SARS-CoV-2 replication and subsequent cytokine responses that restrict continued virion release. Sci Signal, 2023. 16(782): p. eabq1366.

6. van der Donk, L.E.H., J. Eder, J.L. van Hamme, P.J.M. Brouwer, M. Brinkkemper, A.C. van Nuenen, et al., SARS-CoV-2 infection activates dendritic cells via cytosolic receptors rather than extracellular TLRs. Eur J Immunol, 2022. 52(4): p. 646-655.

7. Li, X.T., M.W. Zhang, Z.Z. Zhang, Y.D. Cao, X.Y. Liu, R. Miao, et al., Abnormal apelin-ACE2 and SGLT2 signaling contribute to adverse cardiorenal injury in patients with COVID-19. Int J Cardiol, 2021. 336: p. 123-129.

8. Mitchell, D.A., S.K. Nair, and E. Gilboa, Dendritic cell/macrophage precursors capture exogenous antigen for MHC class I presentation by dendritic cells. Eur J Immunol, 1998. 28(6): p. 1923-33.

9. Qin, X.Y., Y.L. Zhang, Y.F. Chi, B. Yan, X.J. Zeng, H.H. Li, et al., Angiotensin II Regulates Th1 T Cell Differentiation Through Angiotensin II Type 1 Receptor-PKA-Mediated Activation of Proteasome. Cell Physiol Biochem, 2018. 45(4): p. 1366-1376.

10. Barhoumi, T., F.A. Mansour, M. Jalouli, H.S. Alamri, R. Ali, A.H. Harrath, et al., Angiotensin II modulates THP-1-like macrophage phenotype and inflammatory signatures via angiotensin II type 1 receptor. Front Cardiovasc Med, 2023. 10: p. 1129704.

11. Wu, L., K. Chen, J. Xiao, J. Xin, L. Zhang, X. Li, et al., Angiotensin II induces RAW264.7 macrophage polarization to the M1‑type through the connexin 43/NF‑kappaB pathway. Mol Med Rep, 2020. 21(5): p. 2103-2112.

12. Yamamoto, S., P.G. Yancey, Y. Zuo, L.J. Ma, R. Kaseda, A.B. Fogo, et al., Macrophage polarization by angiotensin II-type 1 receptor aggravates renal injury-acceleration of atherosclerosis. Arterioscler Thromb Vasc Biol, 2011. 31(12): p. 2856-64.

13. Son, S.E. and D.S. Im, Therapeutic effects of candesartan in inflammatory skin disorders by suppressing Th17 differentiation. Int Immunopharmacol, 2023. 124(Pt B): p. 110995.

14. Lu, C., M.A. Amin, and D.A. Fox, CD13/Aminopeptidase N Is a Potential Therapeutic Target for Inflammatory Disorders. J Immunol, 2020. 204(1): p. 3-11.

15. Ghosh, M., B. McAuliffe, J. Subramani, S. Basu, and L.H. Shapiro, CD13 regulates dendritic cell cross-presentation and T cell responses by inhibiting receptor-mediated antigen uptake. J Immunol, 2012. 188(11): p. 5489-99.

16. Reinhold, D., A. Biton, A. Goihl, S. Pieper, U. Lendeckel, J. Faust, et al., Dual inhibition of dipeptidyl peptidase IV and aminopeptidase N suppresses inflammatory immune responses. Ann N Y Acad Sci, 2007. 1110: p. 402-9.

17. Reinhold, D., U. Bank, M. Tager, S. Ansorge, S. Wrenger, A. Thielitz, et al., DP IV/CD26, APN/CD13 and related enzymes as regulators of T cell immunity: implications for experimental encephalomyelitis and multiple sclerosis. Front Biosci, 2008. 13: p. 2356-63.

18. Hu, L., Y. Hayashi, H. Kidoya, and N. Takakura, Endothelial cell-derived Apelin inhibits tumor growth by altering immune cell localization. Sci Rep, 2021. 11(1): p. 14047.

19. Celik, F.S., C.E. Gunes, E. Yavuz, and E. Kurar, Apelin triggers macrophage polarization to M2 type in head and neck cancer. Immunobiology, 2023. 228(2): p. 152353.

20. Li, X.T., M.W. Zhang, Z.Z. Zhang, Y.D. Cao, X.Y. Liu, R. Miao, et al., Abnormal apelin-ACE2 and SGLT2 signaling contribute to adverse cardiorenal injury in patients with COVID-19. Int J Cardiol, 2021. 336: p. 123-129.

21. Li, S., S. Li, Q. Li, F. Liu, W. Liao, L. Yu, et al., Increased Neuromedin B is Associated with a Favorable Prognosis in Glioblastoma. Front Biosci (Landmark Ed), 2023. 28(3): p. 54.

22. Zhou, S., E.N. Potts, F. Cuttitta, W.M. Foster, and M.E. Sunday, Gastrin-releasing peptide blockade as a broad-spectrum anti-inflammatory therapy for asthma. Proc Natl Acad Sci U S A, 2011. 108(5): p. 2100-5.

23. Oliveira, P.G., R. Grespan, L.G. Pinto, L. Meurer, J.C. Brenol, R. Roesler, et al., Protective effect of RC-3095, an antagonist of the gastrin-releasing peptide receptor, in experimental arthritis. Arthritis Rheum, 2011. 63(10): p. 2956-65.

**
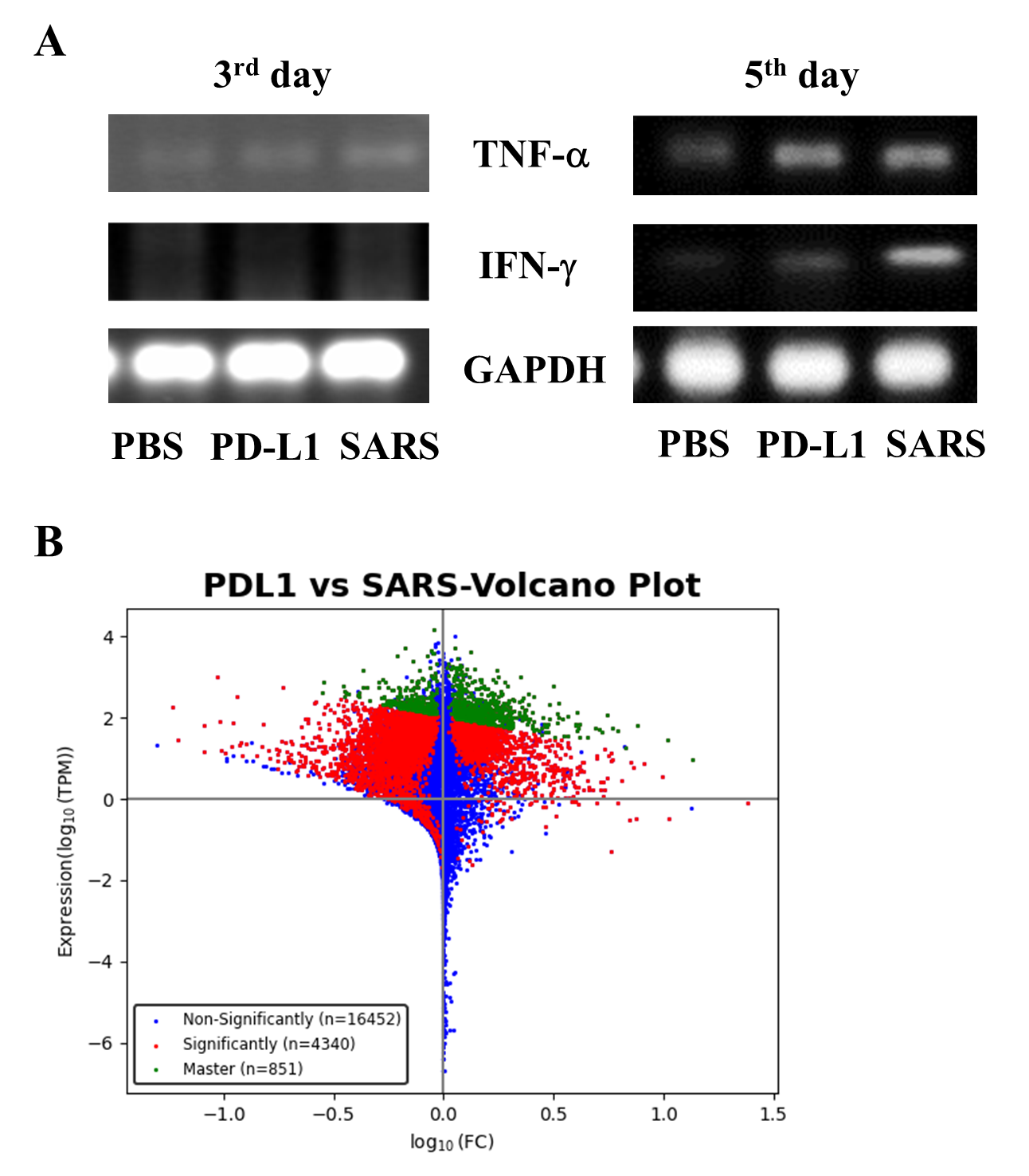
**

**Figure S1. RT-PCR analysis of mouse splenocytes.** (A) RT-PCR evaluated immune activities of PD-L1 peptides or PD-L1-SARS peptides, such as TNF-α, IFN-γ, and GAPDH, on the 3^rd^ and 5^th^ days. A representative image of gel electrophoresis was shown. (B) Gene expression was further analyzed and selected by *t*-tests and ANOVA. The differential expression genes (DEGs) with significant differences (*P*<0.01) through *t*-test, and 851 genes represented the top 70% of the DEGs (green spots).

**
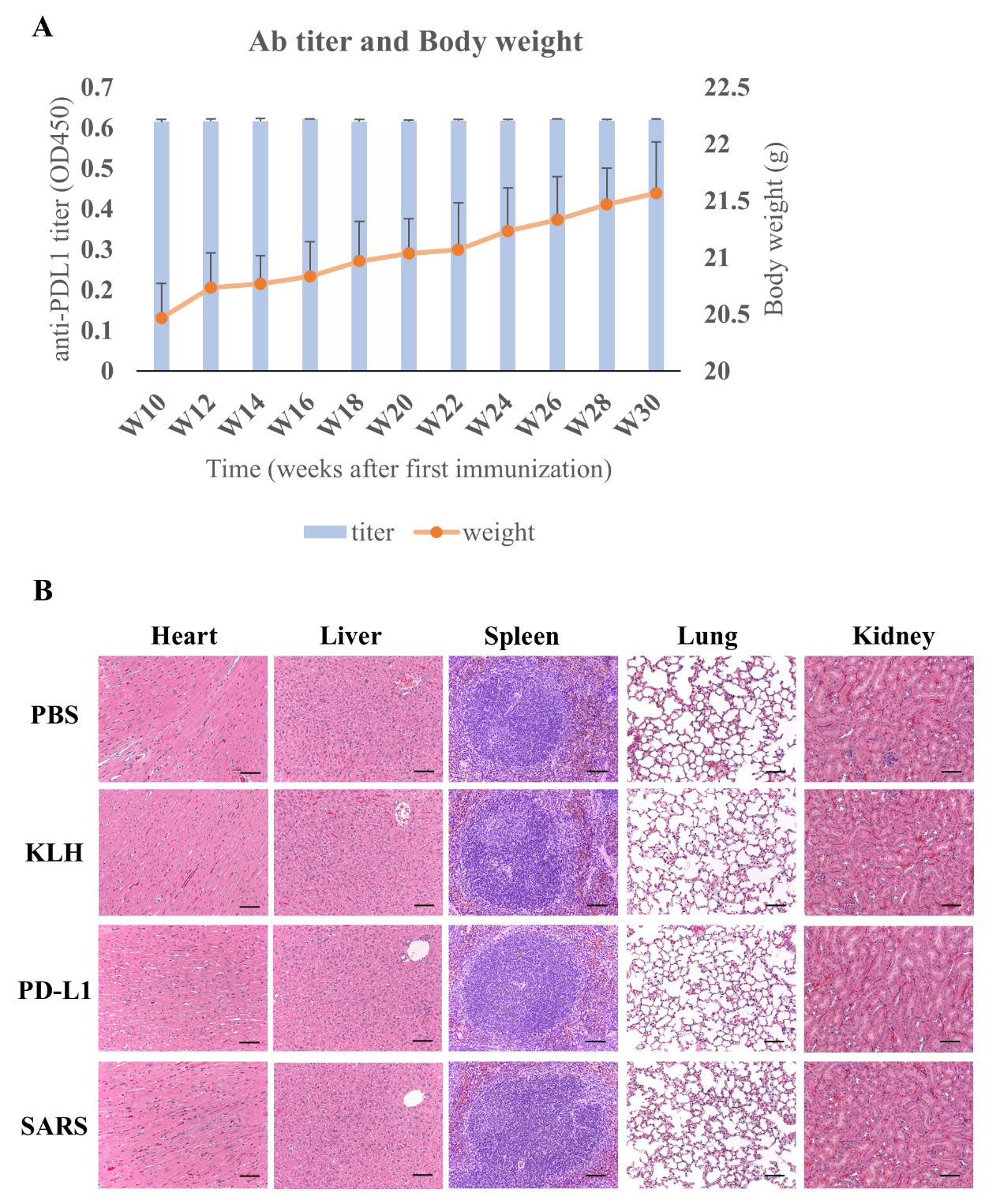
**

**Figure S2. The safety of the PD-L1-SARS peptide vaccine.** (A) The body weights and anti-PD-L1 antibody titers of mice that were vaccinated for 30 weeks were monitored. (B) H&E staining revealed no visible differences in the important organs of the mice (Scale bar of 50 μm at 200× magnification).


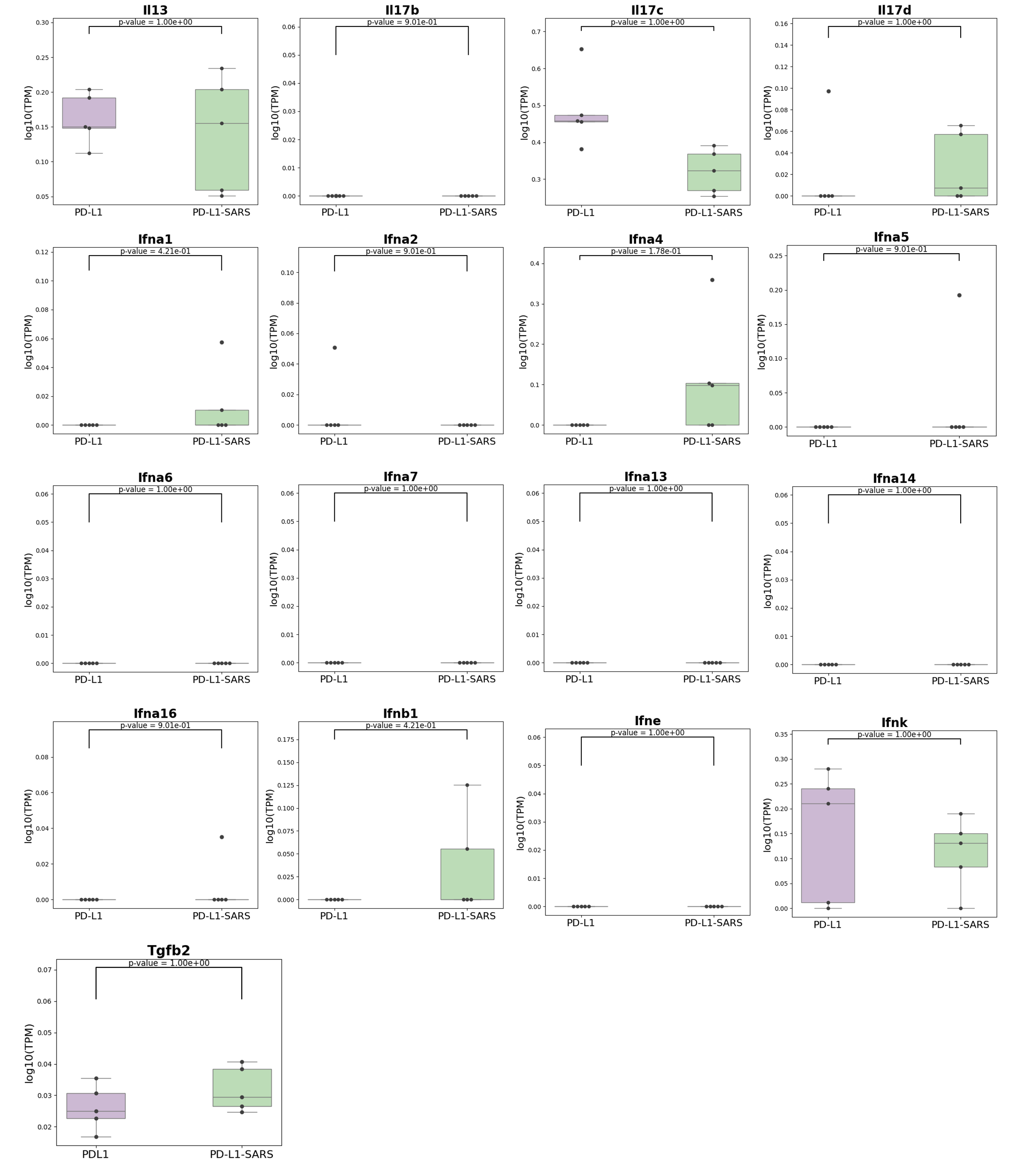


**Figure S3. Cytokine expression according to RNA-seq analysis.** A total of 851 DEGs between the PD-L1 with PD-L1-SARS peptide groups were analyzed, and IL-13, IL-17, IFN, and TGF-β2 cytokine expression was shown.

**
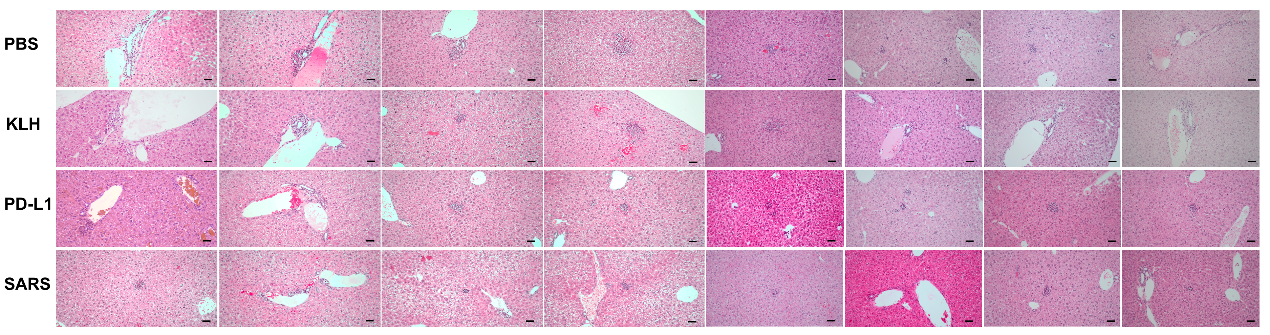
**

**Figure S4. Representative liver H&E sections across treatment groups.** Representative H&E stained liver sections are shown for PBS, KLH, PD-L1, and PD-L1-SARS groups, each panel derived from different animals within the respective group (Scale bar of 25 μm at 200× magnification).


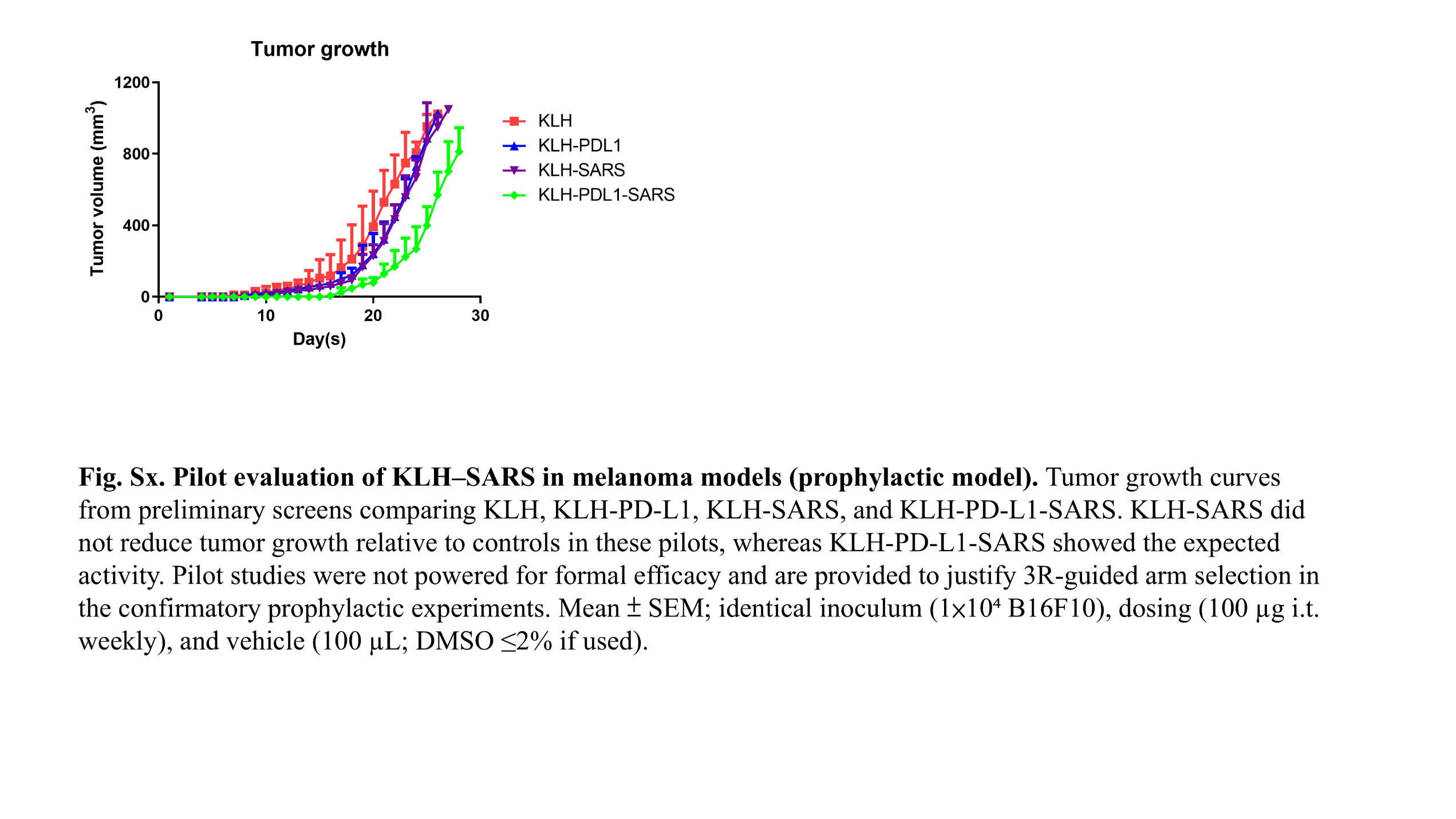


**Figure S5. Pilot evaluation of KLH–SARS in melanoma models (prophylactic model).** Tumor growth curves from preliminary screens comparing KLH, KLH-PD-L1, KLH-SARS, and KLH-PD-L1-SARS. KLH-SARS did not reduce tumor growth relative to controls in these pilots, whereas KLH-PD-L1-SARS showed the expected activity. Pilot studies were not powered for formal efficacy and were provided to justify 3R-guided arm selection in the confirmatory prophylactic experiments. Mean ± SD (n=4); identical inoculum (1×10⁴ B16F10), dosing (100 µg s.c. weekly), and vehicle (100 µL; DMSO ≤2% if used).


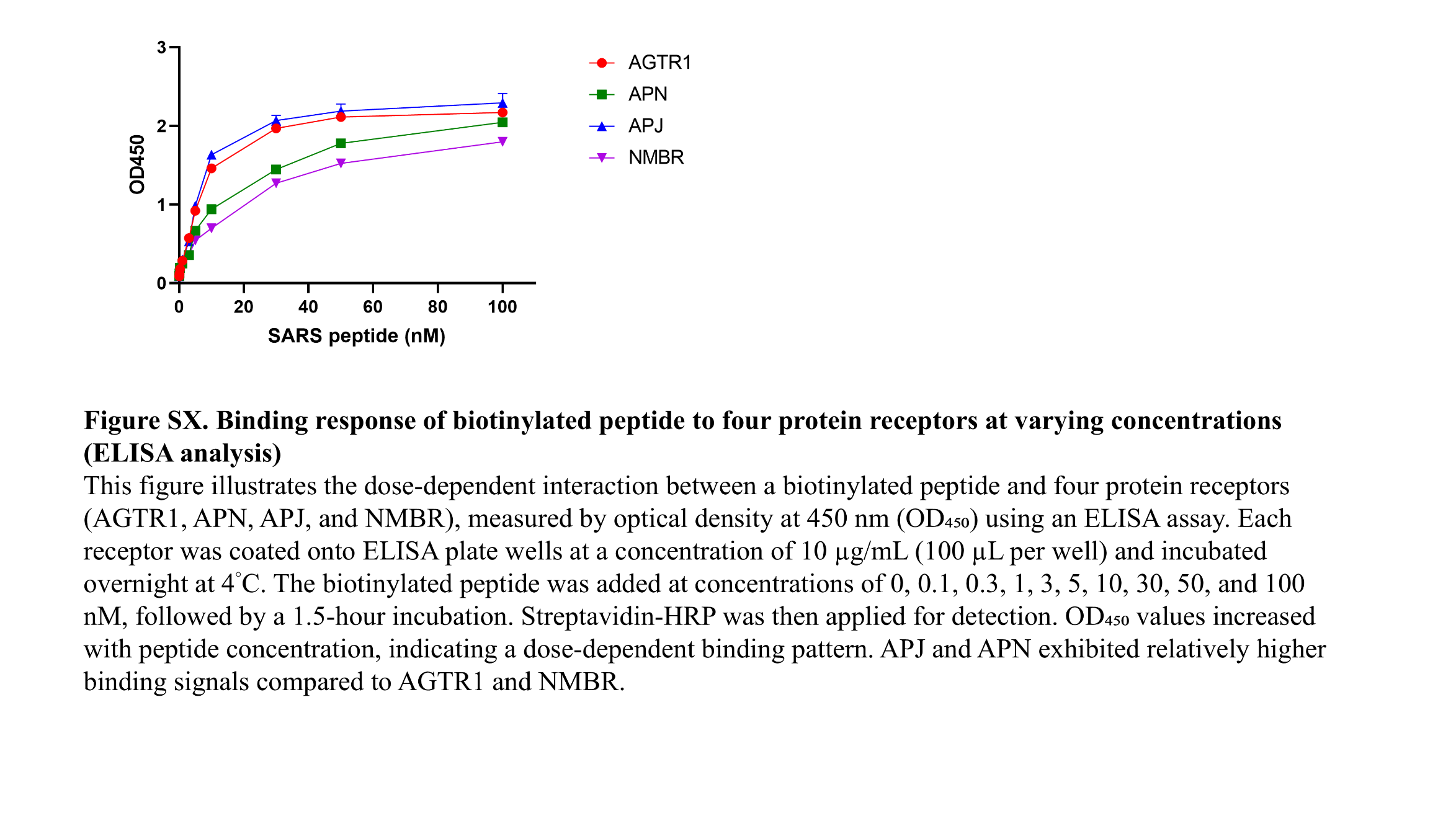


**Figure S6. Binding response of biotinylated peptide to four protein receptors at varying concentrations (ELISA analysis).** This figure illustrates the dose-dependent interaction between a biotinylated peptide and four protein receptors (AGTR1, APN, APJ, and NMBR), measured by optical density at 450 nm (OD₄₅₀) using an ELISA assay. Each receptor was coated onto ELISA plate wells at a concentration of 10 µg/mL (100 µL per well) and incubated overnight at 4°C. The biotinylated peptide was added at concentrations of 0, 0.1, 0.3, 1, 3, 5, 10, 30, 50, and 100 nM, followed by a 1.5-hour incubation. Streptavidin-HRP was then applied for detection. OD₄₅₀ values increased with peptide concentration, indicating a dose-dependent binding pattern. APJ and APN exhibited relatively higher binding signals compared to AGTR1 and NMBR.
